# Myofibroblastic CAF and Malignant Ductal Cell Crosstalk Drives Epithelial–Mesenchymal Transition and Progression in Pancreatic Ductal Adenocarcinoma via THBS2-SDC/Integrin Axes

**DOI:** 10.3390/ijms27156951

**Published:** 2026-08-02

**Authors:** Zhonglu Ren, Zhuangchang Li, Jie Wang, Yuchen Liu, Lidan Chen, Yuxin Su, Limin Zhao, Xi Liu

**Affiliations:** 1 School of Medical Information and Engineering, Guangdong Pharmaceutical University, Guangzhou 510006, China; renzhonglu@gdpu.edu.cn (Z.R.); 2112443010@stu.gdpu.edu.cn (Z.L.); 2112545027@stu.gdpu.edu.cn (J.W.); 13533747960@163.com (Y.L.); 15019754496@163.com (L.C.); 13310889973@163.com (Y.S.); 2 Guangdong Province Precise Medicine Big Data of Traditional Chinese Medicine Engineering Technology Research Center, Guangzhou 510006, China; 3 School of Chemistry and Chemical Engineering, Guangdong Pharmaceutical University, Zhongshan 528458, China

**Keywords:** myCAFs, epithelial–mesenchymal transition, THBS2-SDC/integrin axes, PDAC, multi-omics analysis

## Abstract

Pancreatic ductal adenocarcinoma (PDAC) is a highly lethal malignancy with a five-year survival rate below 10%. Cancer-associated fibroblasts (CAFs) promote epithelial–mesenchymal transition (EMT) and metastasis, yet the specific CAF subtypes and molecular axes driving PDAC progression remain incompletely understood. Here, using multi-omics data from PDAC samples, we identified a malignant ductal subpopulation, termed Ductal-T0, characterized by the highest EMT activity and prominent acquisition of myofibroblastic CAF (myCAF)-like transcriptional programs. Computationally, we predicted that myCAF-secreted *THBS2* and *FN1* engage the ITGA3/ITGB1/SDC1/SDC4 receptor axes in Ductal-T0 cells, which could activate TNF, NF-κB, TGF-β, and PI3K-AKT-signaling pathways to promote EMT. Pseudotime trajectory and velocity analyses suggested that Ductal-T0 cells exhibited the highest propensity to acquire myCAF-like features among all ductal subpopulations. Survival analysis revealed that an increased proportion of Ductal-T0 cells and elevated abundance of THBS2-ITGA3/ITGB1 and THBS2-SDC1 ligand–receptor pairs were significantly associated with poor prognosis. Spatial transcriptomics further revealed that myCAFs and Ductal-T0 cells co-localized at the tumor margin, which may contribute to reduced immune cell presence via dense extracellular matrix (ECM) barrier formation—a computationally inferred model of EMT-associated immune exclusion and metastatic progression—and identify *THBS2* as a promising candidate for future therapeutic investigation to disrupt CAF–tumor crosstalk in PDAC.

## 1. Introduction

Pancreatic ductal adenocarcinoma (PDAC) is one of the most aggressive and lethal malignancies and is the seventh leading cause of cancer-related mortality worldwide [1,2]. Its clinical presentation is often insidious, with a substantial proportion of patients presenting metastatic disease at diagnosis [3], contributing to a five-year survival rate below 10% [1,2,4]. Elucidating the fundamental mechanisms driving PDAC progression is therefore critical for improving patient outcomes.

Epithelial–mesenchymal transition (EMT) plays a pivotal role in promoting tumor progression, invasion, and distant metastasis and is characterized by the loss of epithelial polarity coupled with the acquisition of a mesenchymal phenotype [5]. EMT activation is tightly regulated by diverse signaling pathways and extracellular factors within the tumor microenvironment (TME), such as TGF-β, BMP, and hypoxic signals [5,6,7]. These signals converge on key intracellular pathways, including TGF-β, Wnt, HIF-1α, Notch, PI3K-AKT, and integrin signaling, which induce transcription factors, such as TWIST1/2 and ZEB1/2. These factors in turn suppress epithelial markers (e.g., *CDH1* and *TJP1*) while promoting mesenchymal markers (e.g., *VIM* and *FN1*) [6,7].

PDAC is typically classified as an ‘immune-cold’ tumor, characterized by a complex composition of diverse cell types and extracellular matrix components [8,9,10]. Cancer-associated fibroblasts (CAFs), as integral components of the PDAC stroma, establish a pro-tumorigenic microenvironment through several mechanisms. These include remodeling of the extracellular matrix (ECM) and increased collagen deposition, which collectively impair drug delivery, limit immune cell infiltration, and induce EMT [4,11,12,13,14,15]. Among the CAF-derived factors implicated in this process, *THBS2* and *FN1* have been identified as critical molecules that facilitate metastasis through EMT-signaling activation [16,17]. Current evidence suggests that *THBS2* serves as a highly specific diagnostic biomarker for PDAC, with its expression level significantly correlating with patient prognosis [18,19,20,21]. Although CAFs are recognized as major contributors to cancer progression by promoting invasion, metastasis, and drug resistance, the precise mechanisms by which they coordinate ECM remodeling to drive PDAC progression and EMT remain unclear.

As pivotal ECM receptors, syndecans (SDC1, SDC4) and integrins (ITGA3, ITGB1) are intimately linked to the initiation of EMT and aggressive tumor phenotypes [22,23]. *SDC1* expression correlates with canonical EMT markers, whereas *SDC4* promotes TGF-β1-induced EMT via *SNAI1* [24,25]. Concurrently, the ITGA3/ITGB1 heterodimer activates the FAK/PI3K/AKT/mTOR axis, directly fueling tumor proliferation, metastasis, and EMT [26]. *ITGA3* overexpression serves as an independent prognostic biomarker in pancreatic cancer [25,27]. Collectively, these four receptors converge on major oncogenic pathways to orchestrate EMT-associated tumor progression.

In recent years, CAFs have been identified as pivotal contributors to EMT and tumor metastasis [28]. They facilitate EMT and enhance cancer cell invasion by secreting ECM components and chemokines [29,30]. Emerging evidence suggests that malignant epithelial cells can transdifferentiate into CAFs through EMT, with *SERPINF1^+^* fibroblasts potentially originating from this process [31,32,33,34]. Lin et al. demonstrated that EMT^+^ tumor cell populations in PDAC are associated with increased invasiveness; however, the EMT trajectory and specific CAF subtypes that facilitate metastasis remain to be fully defined [35]. Motivated by the potential of malignant ductal cells in PDAC to undergo EMT and adopt CAF-like traits, this study aimed to identify the key CAF subsets driving EMT, delineate their cellular trajectories, and uncover the EMT-associated molecular axes governing PDAC progression.

In this study, we identified a malignant ductal subpopulation with EMT potential, referred to as Ductal-T0, and explored the molecular pathways by which its interaction with myofibroblastic CAFs (myCAFs) is associated with EMT. We propose the following hypothesis: myCAFs, as crucial ECM-secreting cells, release stromal proteins, including THBS2 and FN1. These proteins are predicted to engage highly expressed receptors ITGA3, ITGB1, SDC1, and SDC4 on the membrane of malignant ductal cells, particularly within the Ductal-T0 subpopulation, which exhibits elevated basal/EMT activity. This interaction establishes a distinct FN1/THBS2-(ITGA3+ITGB1)/SDC1/SDC4 ligand–receptor axes, which is inferred to trigger multiple key signaling pathways, including TNF, NF-κB, TGF-β, and PI3K-AKT. This crosstalk may not only drive EMT in Ductal-T0 cells to induce myCAF-like features, but also accelerate malignant progression and metastasis in PDAC. Through the integration of single-cell and spatial transcriptomic analyses, we showed that THBS2 and FN1 are primarily secreted by myCAFs and exhibit significant spatial co-localization with malignant ductal cells.

## 2. Results

### 2.1. Construction of Single-Cell Transcriptomic Atlas of PDAC

**Figure 1:**
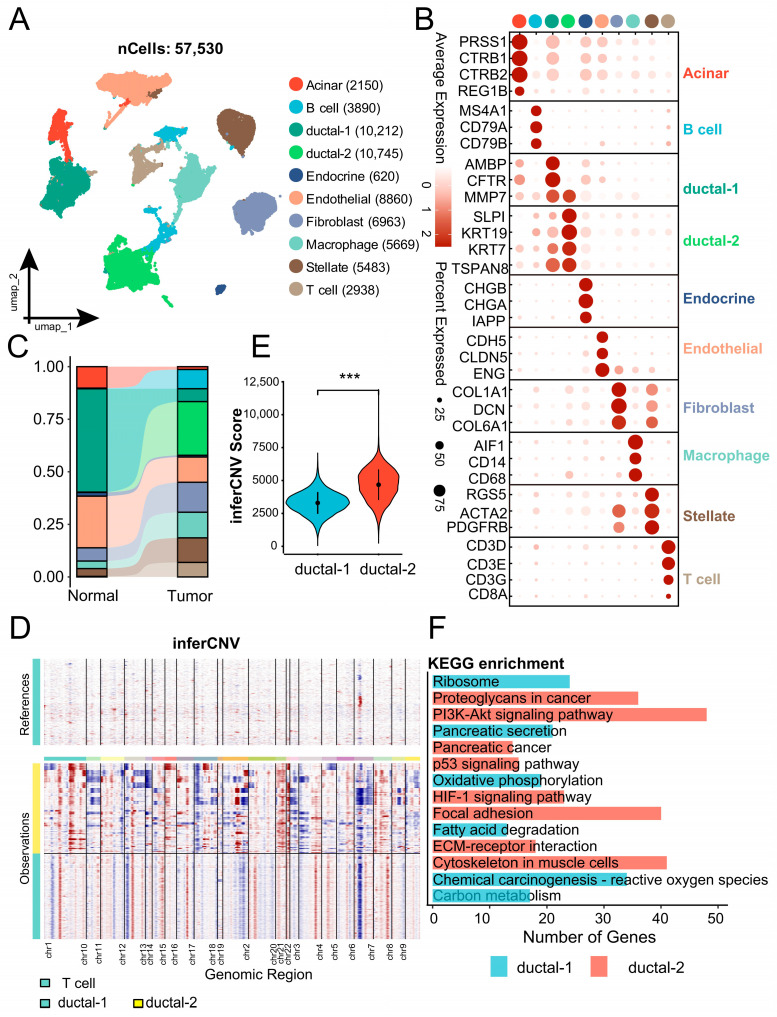
Single-cell landscape defines major cell populations in PDAC. (**A**) UMAP visualization of 57,530 single cells from normal and tumor samples, colored by 10 major cell types. (**B**) Bubble plot showing known marker genes across cell types. (**C**) Bar plot showing the proportions of 10 major cell types in normal and tumor samples. (**D**) Chromosome-level heatmap of inferred copy number variations (CNVs) in ductal cell types. Gains (red) and losses (blue) were inferred by averaging gene expression across specified chromosomes. The colored bands represent different chromosomes. (**E**) Violin plot of CNV scores calculated by InferCNV across different cell types (pairwise two-sided Wilcoxon rank-sum test, *** *p* < 0.001). (**F**) KEGG enrichment analysis of differentially expressed genes among ductal cell subgroups (one-tailed hypergeometric test with Benjamini–Hochberg FDR correction).

To comprehensively characterize the cellular heterogeneity and understand the tumor microenvironment landscape of PDAC, we integrated single-cell RNA-seq data from 35 samples, comprising 24 primary PDAC samples and 11 normal pancreatic ductal tissues. To mitigate potential batch effects across the samples, Harmony was applied for data integration and batch correction. Comparison of the clustering results before and after correction confirmed that batch effects were effectively reduced while biological variation was preserved (Supplementary Figure S1A,B). Following quality control and integration, 57,530 cells were retained and 39 cell clusters were identified for downstream analysis (Supplementary Figure S1C,D). We identified 10 major cell types based on known markers, including acinar cells, B cells, T cells, endocrine cells, endothelial cells, stellate cells, fibroblasts, macrophages, and two types of ductal cells (ductal-1 and ductal-2) (Figure 1A,B). At the cell proportion level, Acinar cells, endothelial cells, and ductal-1 cells were higher in the normal samples, whereas ductal-2 cells, immune cells, fibroblasts, and stellate cells exhibited significantly higher in the PDAC samples (Figure 1C). InferCNV analysis revealed that ductal-2 cell exhibited widespread copy number deletions and amplifications (Figure 1D), with a significantly elevated average CNV score compared to ductal-1 cell (Figure 1E). Functional enrichment analysis revealed that tumor-associated pathways, such as pancreatic cancer, proteoglycans in cancer, focal adhesion, ECM–receptor interaction, PI3K-Akt signaling pathway, and HIF-1 signaling pathway were enriched in ductal-2 cell, whereas normal pancreatic pathways were enriched in ductal-1 cells (Figure 1F). These results suggest that ductal-2 cells are a malignant cell subtype.

### 2.2. Distinct Subgroup in Malignant Ductal Cells Shows EMT Activation

**Figure 2:**
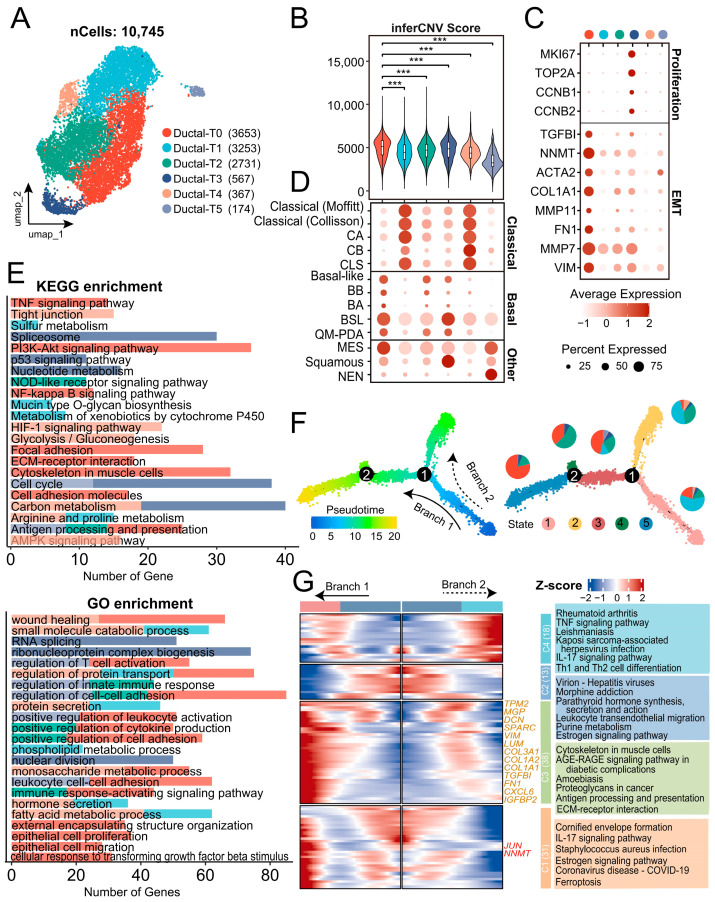
A malignant ductal subgroup exhibits active EMT programs. (**A**) UMAP visualization of 10,745 malignant ductal cells, colored by cell subpopulations. (**B**) Violin plot of CNV scores calculated by InferCNV across malignant ductal cell subpopulations (pairwise two-sided Wilcoxon rank-sum test, *** *p* < 0.001). (**C**) Bubble plot showing average expression of selected genes across cell subpopulations. (**D**) Bubble plot displaying signature scores of PDAC tumor cell subpopulations, colored by gene set scores. (**E**) KEGG (top) and GO (bottom) enrichment analysis of differentially expressed genes among malignant ductal cell subpopulations (one-tailed hypergeometric test with Benjamini–Hochberg FDR correction). (**F**) Differentiation trajectory of malignant ductal cells. Pie charts show the proportions of malignant ductal cell subpopulations in each state. (**G**) Expression dynamics of pattern genes (C1–C4) along pseudotime (left), with corresponding KEGG pathway enrichment results shown on the right. Genes displayed represent the intersection of the Hallmark-EMT gene set and differentially expressed genes.

The ductal-2 cells (10,745 cells) were further divided into 6 subgroups (Ductal-T0, -T1, -T2, -T3, -T4, and -T5) based on clustering analysis after dimensionality reduction (Figure 2A). The cells in the Ductal-T0 subgroup exhibited the highest CNV score (Figure 2B). By comparing gene expression levels, we found that these specific gene sets could be used to distinguish cells in subgroups (Table S1). Notably, Ductal-T0 subgroup cells significantly expressed EMT-related genes, such as *TGFBI*, *COL9A3*, and *CXCL6* (Supplementary Figure S2A), which is further supported by the specific expression of EMT markers (*NNMT*, *COL1A1*, *FN1 MMP7*, and *VIM*) (Supplementary Figure S2B), whereas T3 subgroup cells showed cell proliferation status with the high expression levels of proliferation markers *MKI67*, *TOP2A* and cell cycle markers *CCNB1* and *CCNB2* (Figure 2C). According to the previously established “classic/basal-like” ductal classification system [36], we found that Ductal-T0 was defined as the basal-like (BSL) subtype with the highest mesenchymal score (MES); Ductal-T1 and -T4 belonged to the classic type; Ductal-T3 exhibited squamous characteristics; and Ductal-T5 was the neuroendocrine-like (NEN) subtype. In the Ductal-T2 subgroup, a minority of cells exhibited high expression of BSL markers, while most cells showed low expression of MES markers, suggesting that these cells may span two distinct states (Figure 2D). A higher proportion of cells in the ductal-1, Ductal-T1, and Ductal-T4 subgroups was linked to a favorable prognosis, whereas a higher cellular proportion in the Ductal-T0 and Ductal-T3 subgroups was significantly associated with a poor prognosis. No significant correlation was observed between Ductal-T2 cells and prognosis. (Supplementary Figure S2C).

Functional analysis based on upregulated genes in ductal-2 cell subgroups revealed that Ductal-T0 was significantly enriched in pathways involved in EMT, tumor invasion and metastasis, including focal adhesion, cytoskeleton in muscle cells, ECM–receptor interaction, NF-κB, TNF, and PI3K-Akt signaling pathways. We also observed that the functions of genes specific to Ductal-T0 were mainly related to cell adhesion, cytokine production, epithelial cell proliferation and migration, and cellular response to TGF-β stimulus (Figure 2E). GSEA further confirmed that EMT, angiogenesis, TGF-β, and TNF-α signaling via NF-κB pathways were activated in Ductal-T0 cells (Supplementary Figure S2D).

Two major differentiation trajectories (route 1 and 2) and five cell states (S1–S5) were identified, both rooted from Ductal-T1 cells, which dominate the S1 and S5 states. The route 1 trajectory was bifurcated into either S3 or S4 state. An increased proportion of Ductal-T0 cells along this trajectory was observed, and they finally became the predominant subgroup in the S3 state (Supplementary Figure S2E). In the S4 state, a majority of the cells were occupied by Ductal-T2 cells. In contrast, the route 2 trajectory branched towards cell state S5, which is mainly comprised by Ductal-T1 and Ductal-T2 cells (Figure 2F). Branch expression analysis modeling (BEAM) results identified four distinct patterns of gene expression along pseudotime. In branch 1, the terminal state of differentiation was Ductal-T0 cells, wherein genes within the C3 cluster demonstrated increasing expression levels, accompanied by the emergence of high expression of CAF-related (*COL1A1*, *COL1A2*, and *DCN*) and EMT-related markers (Supplementary Figure S2F). Gene expression in the C2 cluster was downregulated, whereas the C1 cluster showed variations in expression but eventually reached a state of high expression (Figure 2G). In contrast, branch 2 exhibited a terminal state of differentiation in Ductal-T1 and Ductal-T2 cells (state S5). In the C4 cluster, genes showed increased expression along this trajectory, whereas gene expression in the C1 and C2 clusters was downregulated. The C3 cluster displayed variations in gene expression but ultimately reached a low expression state.

In summary, our findings indicate that route 1 delineates the classical-to-basal differentiation trajectory of malignant ductal cells, with alterations in gene expression within the C3 cluster serving as pivotal factors. The enriched pathways suggest that ductal cells acquire capability of migration and the potency to interact with the stromal cells. Ultimately, Ductal-T0 (state S3) presents a basal-like phenotype characterized by elevated EMT activity and invasiveness. Conversely, route 2 may represent the classical differentiation trajectory of malignant ductal cells, wherein the alternating high expression of genes in the C1 and C3 clusters during differentiation collectively facilitates the activation of immune- and inflammation-related pathways in the C4 cluster, such as the TNF signaling pathway, Th1 and Th2 cell differentiation, and the IL-17 signaling pathway. Consequently, the cells in state S5 exhibited a classical-like phenotype with immune-hot and inflammatory activities.

### 2.3. Heterogeneity of Cancer Associated Fibroblasts in the PDAC

**Figure 3:**
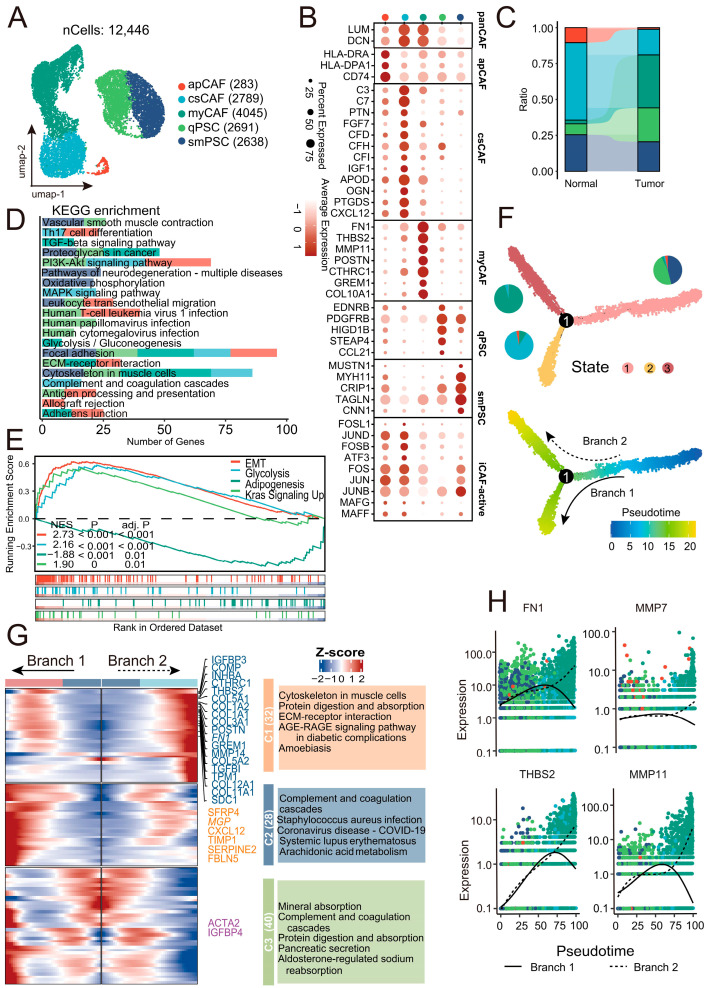
Functional diversity of cancer-associated fibroblasts. (**A**) UMAP visualization of 12,446 cells from normal and tumor samples, annotated into 5 subpopulations and colored by subpopulation. (**B**) Bubble plot showing expression levels of marker and signature genes across 5 subpopulations. (**C**) Bar plot showing the proportions of CAF subpopulations in normal and tumor samples. (**D**) KEGG pathway enrichment analysis of differentially expressed genes in 5 subpopulations (one-tailed hypergeometric test with Benjamini–Hochberg FDR correction). (**E**) GSEA showing significantly upregulated Hallmark gene set pathways in the myCAF subpopulation (permutation-based significance with Benjamini–Hochberg FDR correction). (**F**) Pseudotime analysis of differentiation trajectory. Pie charts show the proportions of different cell subpopulations in each state. (**G**) Expression dynamics of pattern genes (C1–C3) along pseudotime (left), with corresponding KEGG pathway enrichment results shown on the right. Genes displayed represent the intersection of the Hallmark-EMT gene set and differentially expressed genes. (**H**) Expression trends of myCAF-related genes along pseudotime.

We analyzed the stromal compartment in the TME of PDAC, with an emphasis on fibroblasts and pancreatic stellate cells (PSCs), because they play crucial roles in the development and treatment of PDAC [37]. CAFs have been previously divided into inflammatory CAFs (iCAFs), myofibroblastic CAFs (myCAFs), antigen-presenting CAFs (apCAFs), and complement-secreting CAFs (csCAFs) in the pancreatic tumor microenvironment [38,39]. In this study, we classified fibroblasts into three subclusters: apCAFs, csCAFs, and myCAFs, and identified two previously described PSCs: smooth muscle (smPSCs) and quiescent PSCs (qPSCs) (Figure 3A and Figure S3A). *ACTA2* is highly expressed in both CAFs and PSCs, while *FAP* is expressed in both myCAFs and csCAFs. Therefore, relying solely on these classic markers is insufficient to distinguish distinct CAF subtypes. To more accurately define and characterize CAF populations, we utilized *LUM* and *DCN* as pan-CAF markers, and additionally employed *COL10A1, FN1*, *THBS2*, *MMP11*, *POSTN* and *CTHRC1* to more comprehensively define the myCAFs identity (Figure 3B and Figure S3B). Furthermore, *COL10A1* (ECM remodeling), *FN1* (EMT-related), *THBS2* (myCAF marker in mouse KPC model [40]) were also enriched in myCAFs (Figure 3B and Figure S3B). csCAFs were identified by complement genes (*C3*, *C7* and *CFD*) as well as other markers. *CXCL12* was specifically upregulated in csCAFs, which is an important ligand for communication with immune cells (B cells, T cells, and myeloid cells), and can serve as a novel csCAF marker. apCAFs are a rare cell type that is predominantly present in normal pancreatic tissue. The proportion of csCAFs decreased in tumor samples compared to normal samples. Conversely, a significantly increased proportion of myCAFs was observed in tumor samples (2.57% in normal and 36.78% in tumor samples), showing a distribution similar to Ductal-T0 cells (Figure 3C).

There were 1053 upregulated genes screened in myCAFs compared to other cell types (Supplementary Figure S3C and Supplementary Table S2), and enriched pathways related to cancer-promoting microenvironment program, such as proteoglycans in cancer, ECM–receptor interaction, cytoskeleton in muscle cells, focal adhesion, adherens junction, and TGF-β signaling pathways. The upregulated genes in csCAFs were enriched in pathways regulating immune and inflammatory responses within tumors, such as complement and coagulation cascades and the MAPK signaling pathway; those in apCAFs were associated with Th17 cell differentiation and antigen processing and presentation; and those in smPSC cells were linked to vascular smooth muscle contraction and oxidative phosphorylation (Figure 3D). GSEA results revealed that EMT, Glycolysis and KRAS signaling upregulation pathways were highly activated in myCAFs of PDAC (Figure 3E).

PSCs are considered a source of CAFs in PDAC, and there exists potential for transdifferentiation among different CAF subtypes [37,41]. Monocle2 analysis inferred bifurcation differentiation routes into two directions and three cell states (S1–S3). Both trajectory routes were rooted in state S1. Route1 consisted of states S1 and S2, where csCAFs (state S2) transitioned from apCAFs and PSCs (smPSCs and qPSCs). In contrast, CAFs in route 2 developed from apCAFs and PSCs to myCAFs as the differentiation endpoint, which comprising states S1 and S3 (Figure 3F and Figure S3D). The BEAM results showed that in route 1, there was elevated expression of genes in the C2 cluster from the pre-branch to the final state 2 (csCAFs). Complement, immune regulation, and inflammatory response pathways were enriched in this cluster. Route 2 exhibited high expression of genes in the C1 cluster and enriched ECM–receptor interaction and cytoskeleton in muscle cell pathways (Figure 3G). Furthermore, nearly 60% of the genes (19/32) in the C1 cluster were involved in EMT, and such as *FN1*, *THBS2*, and *MMP* family genes were upregulated along this route. (Figure 3H). Although we did not identify the iCAFs subtype, high expression of the AP-1 family (*FOS*, *JUN*) was observed in csCAFs, suggesting that csCAFs possess differentiation potency into iCAFs (Figure 3B) [41].

These results indicated that csCAFs are in an activated state of differentiating into iCAFs and possess the ability to talk to immune cells. myCAFs differentiate into an EMT-activated state with enhanced communication capabilities with tumor cells. An increased proportion of myCAFs in tumor samples supports the hypothesis that Ductal-T0 cells, which exhibit basal-like transcriptional features, may undergo EMT-associated state transitions toward a myCAF-like identity.

### 2.4. Ductal-T0 Cells Exhibit EMT-Associated Transcriptional State Transitions Suggestive of myCAF-like Features

**Figure 4:**
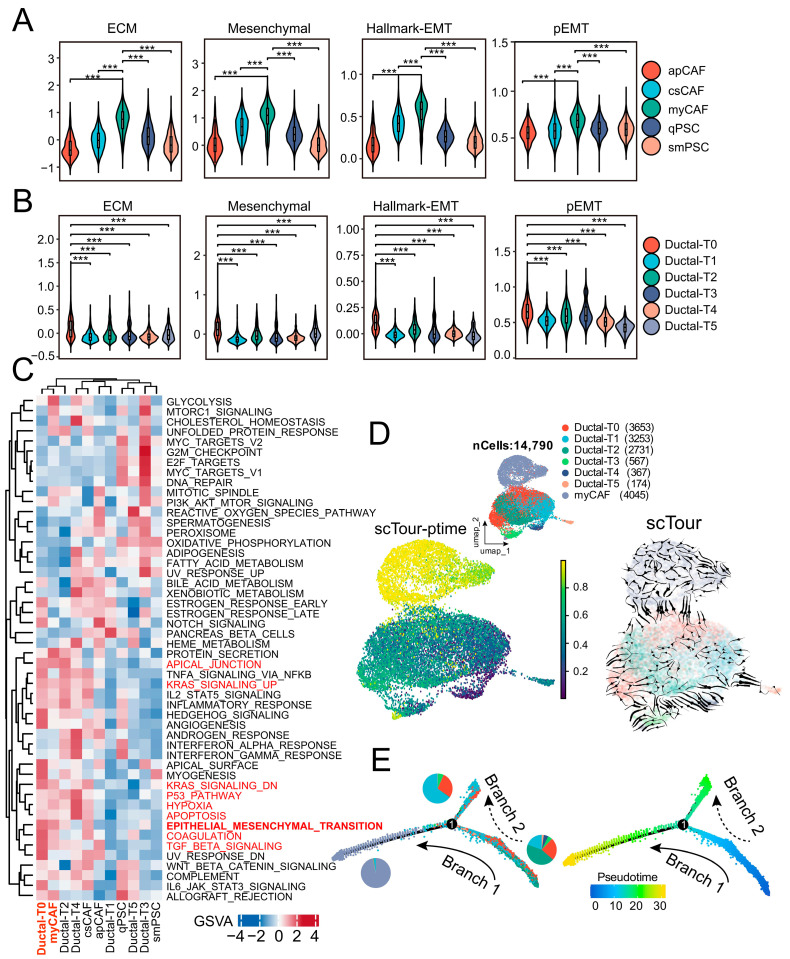
Ductal-T0 cells harbor myofibroblastic differentiation potential. (**A**) Violin plots showing the distribution of scores for ECM, Mesenchymal, Hallmark-EMT, and pEMT gene sets across different CAF subpopulations. (**B**) Violin plots showing the distribution of scores for ECM, Mesenchymal, Hallmark-EMT, and pEMT gene sets across malignant ductal cell subpopulations ((**A**,**B**): pairwise two-sided Wilcoxon rank-sum test, *** *p* < 0.001). (**C**) Heatmap showing GSVA enrichment scores of 50 Hallmark gene sets across CAF subpopulations and malignant ductal cell subpopulations. Pathways shown in red represent those commonly enriched in both Ductal-T0 and myCAFs. (**D**) EMT trajectory inferred by scTour. Left, cell projection colored by pseudotime; right, vector field plot. (**E**) EMT differentiation trajectory inferred by Monocle 2. Pie charts show the proportions of different cell subpopulations in each state.

Based on the gene expression of Ductal-T0 cells and myCAFs, we calculated the invasion, ECM, EMT (invasion and EMT gene sets from MSigDB and pEMT gene set from Barkley [42]), and mesenchymal gene set scores. The results showed that myCAFs had the highest ECM, EMT, and mesenchymal scores compared with other CAFs, as well as a moderate migration score (Figure 4A and Figure S4A). In the malignant ductal cell clusters, the Ductal-T0 cluster exhibited the highest ECM, EMT, mesenchymal, invasion, and migration scores (Figure 4B and Figure S4B). Cluster analysis of malignant ductal cells and CAFs using 50 hallmark gene set scores revealed that Ductal-T0 and -T2 clusters and myCAFs were located in the left branch, with Ductal-T0 and myCAFs being more closely clustered. In addition to the highest EMT scores, Ductal-T0 and myCAFs also showed high similarity in scores for the apical junction, KRAS signaling (UP and DOWN), P53, hypoxia, apoptosis, coagulation, and TGF-β signaling pathways (Figure 4C). Furthermore, in tumor samples from the single-cell RNA-seq dataset, there was a significant positive correlation between the number of Ductal-T0 cells and myCAFs (*R* = 0.343, *p* = 0.0435, Supplementary Figure S4C). These results suggest that Ductal-T0 cells and myCAFs share transcriptional similarity within the TME. However, it is important to note that transcriptional similarity does not necessarily indicate biological identity equivalence or direct lineage conversion.

Furthermore, we integrated all malignant cells (Ductal-T0-T5) and myCAFs (total of 14,790 cells). scTour analysis showed that some Ductal-T0 cells were adjacent to myCAFs in the UMAP space, and velocity streams from Ductal-T0 cells finally flowed to myCAFs, suggesting that these cells may share transcriptional continuity along an inferred EMT-associated trajectory (Figure 4D). A pseudotime developmental tree was constructed, and three branches with malignant ductal cells and myCAFs were identified. The mixed cell population (dominated by Ductal-T0, -T1, and -T2) was located at the initial position of state S1. Some Ductal-T0 cells (110/4188) and all myCAFs were located at the endpoint of pseudotime trajectory, and these Ductal-T0 cells were observed to be earlier than that of myCAFs in state S2 (Figure 4E). Estimation of fate probabilities by CellRank2 also showed that Ductal-T0 cells have 3 possible terminal states (T0_2, T0_3, and T0_5) concentrated at the initial positions of velocity streams by scTour, and finally towards the myCAFs terminal state (Figure S4D). The gene expression pattern across pseudotime showed that a significant reduction in the expression of epithelial markers (*EPCAM*, *KRT19*, and *CDH1*) was accompanied by an increase in EMT-related genes (*COL1A1*, *FN1*, *NNMT*, and *VIM*) and myCAF markers (*ACTA2*, *TAGLN*) in Route 1 (states S1 and S2). The expression of mesenchymal stromal cell markers (*THY1*, *CD44*, and *NT5E*) was also altered correspondingly (Supplementary Figure S4E). Compared with other malignant ductal cell clusters, *CXCL1*, *SFRP2*, and *SERPINF1* were highly expressed in the Ductal-T0 cluster (Supplementary Figure S4F). These genes served as special markers for CAFs subtypes in colorectal cancer (*CXCL1*^+^ myCAFs and *SERP2*^+^ myCAFs) and metastatic liver tumors (*SERPINF1*^+^ fibroblasts), which were confirmed to be derived from the mesenchymal transition of malignant epithelial cells [31]. These findings suggest that Ductal-T0 cells, positioned at the terminal end of the inferred pseudotemporal trajectory, possess mesenchymal and basal-like transcriptional characteristics and show potential to acquire myCAF-like features along inferred trajectories. The observed shared transcriptional features may reflect convergent EMT programs activated in both cell types, whereas, definitive establishment of transdifferentiation requires lineage-tracing experiments.

### 2.5. FN1/THBS2-SDC1/SDC4/(ITGA3+ITGB1) Axes Are Predicted to Promote Communications Between myCAFs and Ductal-T0 Cells

**Figure 5:**
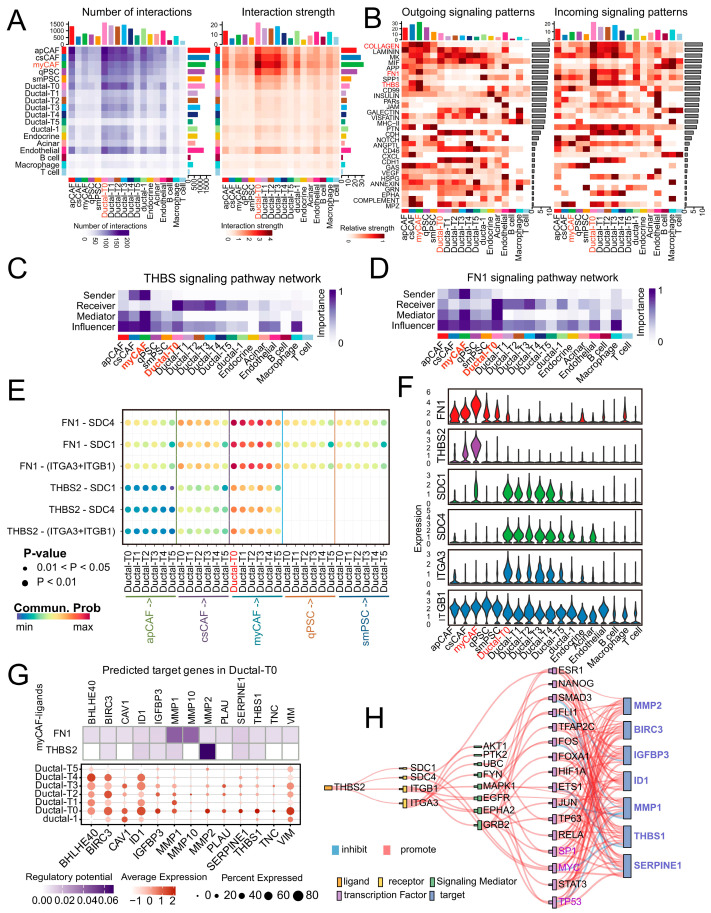
THBS2/FN1-SDC/integrin axes mediate myCAF–Ductal-T0 crosstalk. (**A**) Heatmap showing the number (left) and strength (right) of ligand–receptor interactions between different cell types. (**B**) Heatmap showing outgoing (left) and incoming (right) signaling patterns associated with different cell types. (**C**) Network diagram of the FN1 signaling pathway. (**D**) Network diagram of the THBS signaling pathway. (**E**) Bubble plot showing communication probabilities of key ligand–receptor pairs in FN1 and THBS signaling pathways (CellChat-inferred communication probability assessed by permutation test). (**F**) Violin plots showing differential expression levels of key ligand–receptor pairs in the signaling pathways. (**G**) Heatmap showing the potential activity of two key ligands regulating Ductal-T0 target genes. Bubble plot showing expression levels of target genes. (**H**) Network diagram of the potential signaling pathway through which THBS2 regulates seven target genes. Bold font indicates the top 10 nodes calculated by the MCC algorithm in Cytoscape (v3.9.1).

Based on these observations, we hypothesized that tumor cells interact with CAFs to promote differentiation and cell type transformation. Cell–cell communication analysis was consistent with this notion, showing that the interactions between malignant ductal cells and CAFs were globally much stronger among all cell types, especially myCAFs and Ductal-T0 cells with the highest intensity (Figure 5A). We found that the “COLLAGEN”, “FN1”, and “THBS” signaling pathways were key interactions between myCAFs as signal senders and Ductal-T0 cells as signal receivers in the communication network (Figure 5B–D and Figure S5A,B). Furthermore, FN1-SDC4, FN1-SDC1, FN1-(ITGA3+ITGB1), THBS2-SDC1, THBS2-SDC4, and THBS2-(ITGA3+ITGB1) ligand–receptor pairs exhibited the strongest interactions between myCAFs and Ductal-T0 cells (Figure 5E). In the line with this, ligands secreted by myCAFs, such as *FN1* and *THBS2*, were primarily expressed in myCAFs, whereas receptors of malignant ductal cells, such as *SDC1*, *SDC4*, *ITGA3*, and *ITGB1*, were overexpressed in Ductal-T0 cells (Figure 5F). Notably, the CXCL12-CXCR4 pair between csCAFs and some immune cells (T cells, B cells, and macrophages) was also identified in this study (Supplementary Figure S5C), and this result was consistent with a previous study [41]. We computationally inferred the possible interactions between ligands, receptors, and target genes between myCAFs and Ductal-T0 cells utilizing NicheNet. FN1 and THBS2 were among the top 30 active ligands in myCAFs, and the high interaction potential receptors were consistent with the CellChat results (Supplementary Figure S5D). Furthermore, we demonstrated the target genes that were potentially regulated by *FN1* and *THBS2* in Ductal-T0 cells, such as *BIRC3*, *THBS1*, *SERPINE1*, *MMP1*, *MMP2*, *IGFBP3*, and *ID1* predicted to be regulated by *THBS2*. These genes showed specific expression in Ductal-T0 cells and may act as core genes in EMT. (Figure 5G). Computational inference of the gene regulatory network revealed possible paths from THBS2 as ligand secreted by myCAFs to the final target genes going through receptors in Ductal-T0 cells (Figure 5H). Signaling intermediates or transcription factors in this network were enriched several signaling regulatory pathways of EMT, for example, TGF-β, HIF-1, Ras, and TNF (Supplementary Figure S5E). In the Ductal-T0 cells, SDC1/SDC4 may be the prioritized receptor to interact with THBS2 to influence the TGF-β and phosphorylation cascade signaling pathway, thereby promoting transcription factor-mediated regulation of EMT-related gene expression. Similarly, gene regulatory network from *FN1* to the final target genes was also inferred (Supplementary Figure S5F). Taken together, these computationally derived results suggest that myCAFs and Ductal-T0 cells play a critical role in shaping the TME through the interactions of the *FN1*, *THBS2* signal pathways; furthermore, *THBS2* may serves as an igniter and *SDC1/4* as a booster of the regulatory network to promote EMT progress in malignant ductal T0 cells [43]. Additionally, scRANK results indicated that Ductal-T0 exhibited the highest response to the *SDC1* antagonist among all malignant ductal cells (Supplementary Figure S5G).

### 2.6. THBS2 May Serve as an Essential Ligand Modulating EMT and Is Linked to Poor Prognosis

**Figure 6:**
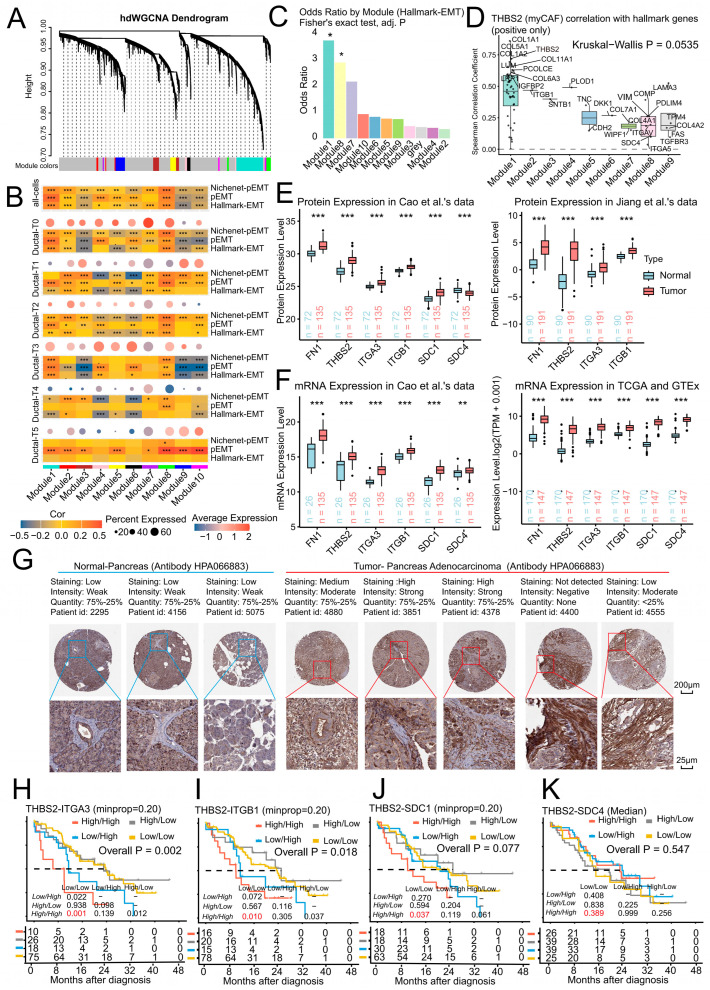
*THBS2* is an essential EMT modulator associated with adverse outcomes. (**A**) Dendrogram of ten gene modules identified by hdWGCNA. (**B**) Module eigengene scores (hMEs) of hdWGCNA gene modules in malignant ductal cell subpopulations and their correlation with EMT features (two-sided Pearson’s correlation coefficient test). (**C**) Bar plot showing the enrichment degree of the Hallmark-EMT gene set across different gene modules (one-sided Fisher’s exact with Benjamini–Hochberg correction, * *adj p* < 0.05; *adj p* ≥ 0.05 are left unmarked). (**D**) Box plot showing the distribution of positive correlation coefficients between THBS2 expression and Hallmark-EMT genes across modules (two-sided Kruskal–Wallis test). (**E**) Box plots showing THBS2 protein abundance in the Cao and Jiang cohorts. (**F**) Box plots showing THBS2 gene expression levels in the Cao cohort and TCGA database ((**E**,**F**): two-sided unpaired *t*-tests). (**G**) Immunohistochemical images of THBS2 in normal pancreas and pancreatic cancer tissue sections from the Human Protein Atlas (HPA, https://www.proteinatlas.org (accessed on 21 May 2026)). Normal tissues: https://v25.proteinatlas.org/ENSG00000186340-THBS2/tissue/pancreas#img (accessed on 21 May 2026); Pancreatic cancer: https://v25.proteinatlas.org/ENSG00000186340-THBS2/cancer/pancreatic+cancer#img (accessed on 21 May 2026). (**H**–**K**) Kaplan–Meier survival curves (Cao cohort protein dataset) showing prognostic differences in patients with high versus low combined expression of THBS2-ITGA3, THBS2-ITGB1, THBS2-SDC1, and THBS2-SDC4 (log-rank test). * *p* < 0.05, ** *p* < 0.01, *** *p* < 0.001; *p* ≥ 0.05 are left unmarked.

To further assess the *THBS2* association with EMT-associated genes, we performed hdWGCNA module analysis on all malignant ductal cells. By selecting the optimal threshold and assessing module interconnectivity, ten non-gray modules were identified (Module 1–10, Figure 6A). The distribution of top 10 kME values and enriched pathways of ten modules revealed their heterogeneity (Supplementary Figure S6A,B). There were three modules significantly enriched in Ductal-T0 cells (Module 1, Module 7, and Module 8), and the correlation matrix with the three EMT gene sets score and enrichment analysis suggested that the Module 1 and Module 8 might be the most relevant for EMT (Figure 6B). Strong correlation was observed among the Module 1, Module4, Module 6, and Module 8 (Supplementary Figure S6C). Therefore, we identified the two modules most closely associated with EMT in Ductal-T0 cells (Module 1 and Module 8). Additionally, genes such as *FN1*, *THBS2*, *SDC1*, and *ITGA3* appeared in Module 1, and *SDC4* appeared in Module 8.

We calculated the correlation coefficients between expression values of *THBS2* in myCAFs and of each gene in Ductal-T0 cells of every module, using Pseudo-bulk method, then we selected positive correlations to obtain relationships between *THBS2* and each module. Overall, Module 1 and Module 8 exhibited a high correlation with THBS2 expression in myCAFs (Supplementary Figure S6D). Both modules were significantly enriched with Hallmark-EMT genes, Module 1 contained 51 EMT-related genes (4.03%, *p* < 3.98 × 10^−12^), while Module 8 contained 10 EMT-related genes (3.98%, *p* < 3.95 × 10^−3^) (Figure 6C). These EMT-related genes showed strong correlations with *THBS2* expression levels (Supplementary Figure S6E). Furthermore, *THBS2* correlated positively with EMT-related genes in Module 1 (86.27%, 44/51; median *R* = 0.46) and Module 8 (80%, 8/10; median *R* = 0.19) (Supplementary Table S3 and Figure 6D). The enrichment of EMT genes and their positive correlation in Module 1 and Module 8 were also confirmed in the other two EMT-related gene sets (Nichenet-pEMT and pEMT, Supplementary Figure S6F,G). These results suggest that *THBS2*, a myCAF-derived ligand, correlates with EMT-associated modules (Module 1 and Module 8), which may drive the acquisition of myCAF-like features in Ductal-T0 cells.

We also investigated gene expression and protein abundance levels of THBS2 and FN1 pathways using TCGA-PAAD (https://portal.gdc.cancer.gov/ (accessed on 1 September 2025)) [44], Cao’s, and Jiang’s datasets. Differential expression analysis was performed on these datasets, and THBS2 and FN1 were upregulated in tumor samples in both RNA-seq and protein array datasets (Supplementary Figure S6H). These genes exhibited significant consistency across different datasets, as well as at both the gene expression levels and protein abundance, such as *THBS2*, *FN1*, *SDC1*, *ITGA3*, and *ITGB1*, which were significantly upregulated in tumor samples compared with normal tissues in both gene expression and protein abundance (Figure 6E,F, Wilcoxon test *p* < 0.05). We confirmed *THBS2* upregulation in tumor tissues using the Human Protein Atlas (HPA) database (https://www.proteinatlas.org (accessed on 21 May 2026)) (Figure 6G) [45,46,47]. We demonstrated that high expression of ligand–receptor pairs, such as THBS2-ITGA3, THBS2-ITGB1, and THBS2-SDC1, was associated with poor prognosis in patients with tumors (Figure 6H–J); however, THBS-SDC4 did not show this trend (Figure 6K). In the FN1 pathway, highly expressed ligand–receptor pairs FN1-ITGA3, FN1-ITGB1, and FN1-SDC1 were also associated with poor prognosis compared to other expression combinations (Supplementary Figure S6I).

### 2.7. myCAFs and Ductal-T0 Cells Co-Localize in a Spatial Niche Characterized by High EMT Activity

**Figure 7:**
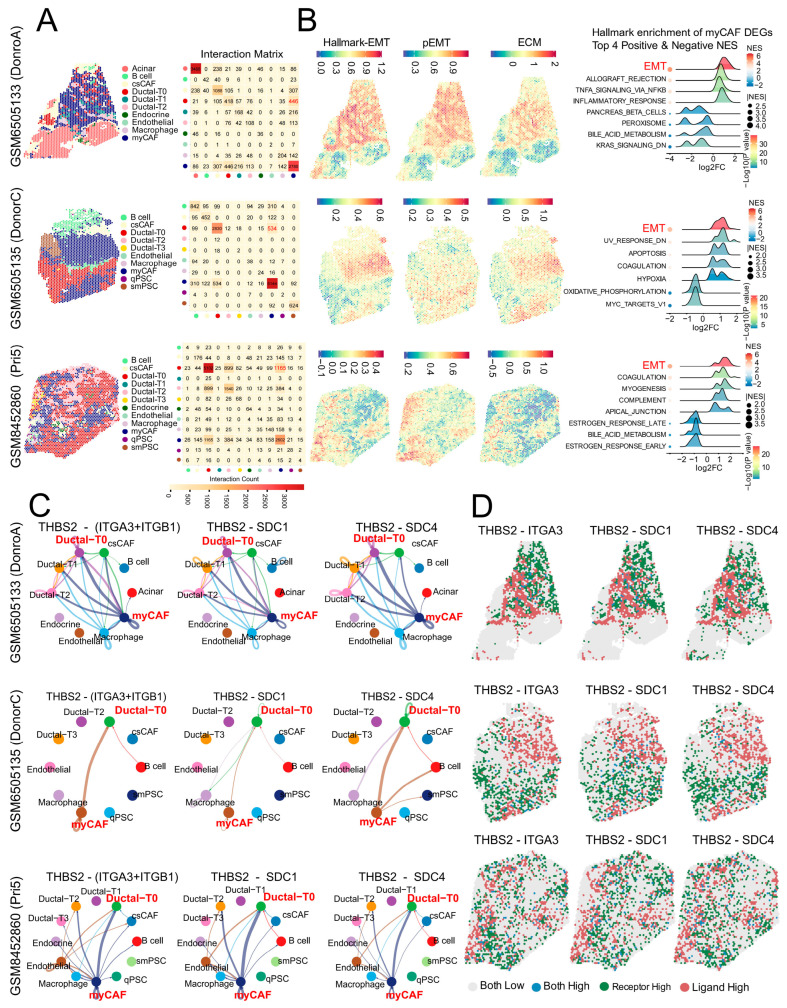
EMT-active niche harbors colocalized myCAFs and Ductal-T0 cells. (**A**) Spatial transcriptomics showing cell types at each spot defined by the dominant cell type proportion; neighborhood matrix heatmap within hexagonal rings surrounding each spot. (**B**) Spatial distribution of Hallmark-EMT, pEMT, and ECM scores on tissue sections; GSEA mountain plot of myCAF differentially expressed genes (permutation-based significance with Benjamini–Hochberg FDR correction). (**C**) The cell–cell interaction network displays the interactions of THBS2 and its receptors within the spatial transcriptome. (**D**) The spatial tissue maps show the colocalization patterns of THBS2 and its receptors.

To investigate the spatial characteristics and functional significance of myCAFs and malignant ductal T0 cells within the TME, we integrated scRNA-seq data for the deconvolution of ST capture locations to predict dominant cell types and ratios. ST data showed that the tumor region was predominantly enriched with Ductal-T0, -T2, and -T3 cells and reduced immune cell presence, while the tumor margins exhibited high-density myCAFs, which showed the strongest spatial colocalization with Ductal-T0 cells. These T0 cells were either encapsulated by myCAFs or showed a distinct interface with myCAFs (Figure 7A and Figure S7A,B). These co-localized regions exhibited EMT activation in Ductal-T0 cells and ECM activation in myCAFs, and the GSEA-enriched pathways further supported these results. Specifically, GSEA revealed that the DEGs in myCAFs were significantly enriched in EMT pathway (Figure 7B). These results suggest that myCAFs may contribute to ECM remodeling at the tumor margin, and that this niche pattern may facilitate intercellular communication between myCAFs and Ductal-T0 cells.

We further evaluated spatial intercellular communication and inferred ligand–receptor interaction pairs, revealing that in four out of five ST samples, this niche exhibited the most frequent interaction between myCAFs as the signal sender and Ductal-T0 cells as the receiver (Supplementary Figure S7C). Analysis of ligand–receptor pair interactions identified the strongest interactions between myCAFs and Ductal-T0 cells in the THBS2-SDC1/SDC4 and THBS2-(ITGA3-ITGB1) pairs, with corresponding spatial co-localization distributions matching their respective cell types (Figure 7C,D and Figure S7A,B). Similarly, we observed stronger interactions between myCAFs and Ductal-T0 cells in the FN1 pathway, in which the ligand–receptor pairs FN1-SDC1/SDC4 and FN1-(ITGA3-ITGB1) were involved (Supplementary Figure S7A,B,D–F).

**Figure 8:**
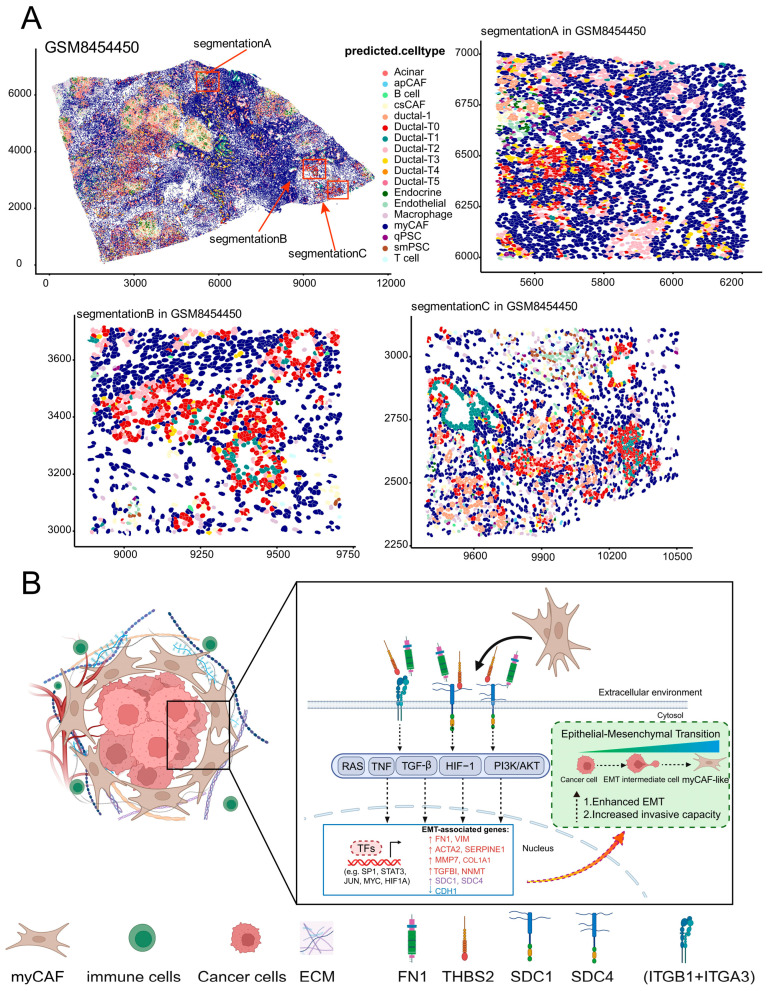
Cellular colocalization of myCAFs with Ductal-T0 cells and ligand–receptor colocalization of THBS2-SDC1. (**A**) Panoramic view of Xenium slices showing major cell types inferred by cellular deconvolution. Three magnified regions display local spatial arrangement and colocalization of cell types. (**B**) Proposed mechanism: myCAF-derived THBS2 and FN1 are predicted to activate EMT in malignant ductal cells via SDC1/SDC4/ITGA3 axes, potentially contributing to EMT progression. Components supported by direct spatial transcriptomic observations (e.g., myCAF–Ductal-T0 co-localization and marker expression) are shown in solid lines. Computationally inferred interactions (e.g., downstream pathway activation, EMT-associated TFs, and EMT transitions) are shown in dashed lines. Created in BioRender. Zhuangchang Li. (2026) https://app.biorender.com/illustrations/canvas-beta/684a95945ad511febe43fb10?slideId=e636c58f-d164-4803-a70e-44d0ee95ea78.

Xenium ST data from three untreated PDAC samples further confirmed the existence of this niche. We observed that myCAFs were significantly enriched in the margins of Ductal-T0 cell tumor region, whereas immune cells, such as T cells, were localized at the tumor–stromal junction, thereby showing a pattern consistent with reduced immune cell infiltration in the core tumor regions (Figure 8A and Figure S8A). Due to the limited number of pre-designed probes (GPL33762) in the GSE274673 dataset, which did not encompass all the ligand–receptor pairs of interest, we detected only high expression of *FN1* and *THBS2* in myCAFs, as well as high expression of *ITGA3* in Ductal-T0 cells (Supplementary Figure S8B).

To further validate the spatial niches of these ligand–receptor pairs, we examined multiple ST samples in the BMS Spatial Portal for Treatment Naïve PDAC database (https://periscopeapps.org/pdac_spatial_portal/ (accessed on 30 April 2026)) [48]. Using *KRT7* to distinguish malignant ductal cells and *FN1* for myCAFs, the findings demonstrated that THBS2-SDC1, THBS2-SDC4, and THBS2-ITGA3 exhibited spatial co-localization distributions. (Supplementary Figure S8C).

## 3. Discussion

Through integrated analysis of scRNA-seq and spatial transcriptomics, this study characterized the phenotypic plasticity of malignant ductal cells and identified a subpopulation with EMT potential. We provided insights into the origin, spatial distribution, EMT trajectory, and niche interactions of this subpopulation. Based on computational inference, we propose the THBS2/FN1-integrin/SDC axes as a putative mechanistic model for EMT. THBS2 and FN1 are predominantly secreted by myCAFs and are enriched at the tumor margin. These myCAF-derived ligands are predicted to interact with their cognate receptors, ITGA3-ITGB1, SDC1, and SDC4, which are highly expressed in Ductal-T0 cells, thereby potentially activating downstream signaling pathways, including TNF, NF-κB, TGF-β, and PI3K-AKT. This signaling cascade may contribute to the EMT program, during which Ductal-T0 cells may acquire further myCAF-like transcriptional features. Concurrently, ST profiling revealed reduced immune cell presence in tumor regions enriched for myCAFs and Ductal-T0 cells, supporting the existence of a distinct stromal niche. We speculate that myCAF-mediated ECM deposition at the tumor margin may contribute to immune cell exclusion; however, as our spatial data demonstrate spatial association rather than functional immune exclusion, this interpretation warrants validation through experimental approaches. A schematic model summarizing these findings is shown in Figure 8B. Collectively, these results contribute to our understanding of how CAFs are associated with PDAC progression.

Ductal-T0 cells occupied an active state of EMT, displayed a basal-like phenotype marked by elevated scores for invasion, migration, and mesenchymal traits, concomitantly exhibiting the greatest potential for transitioning into myCAF-like cells. Consistent with this, Ductal-T0 cells demonstrate the upregulation of *VIM* and basal-like tumor cell signatures [49,50]. Previous studies have established that tumor cells with enhanced EMT, invasiveness, and migratory capacities are more likely to undergo EMT-mediated conversion into CAFs [31]. In this study, Ductal-T0 cells exhibited the highest EMT, migration, and partial EMT (pEMT) scores among all ductal subpopulations, supporting their pronounced tendency to acquire myCAF-like transcriptional features. Furthermore, Ductal-T0 was characterized by gene signatures associated with EMT, angiogenesis, and ECM interactions, together with markedly activated PI3K/Akt and TGF-β signaling pathways. Positioned at the terminal end of the inferred pseudotemporal trajectory, this subpopulation aligns with the characteristics of EMT-associated tumor cells described in multiple independent studies [32,49,51,52]. Accumulating evidence suggests that tumor cells undergoing EMT may acquire basal-like features characterized by enhanced EMT and cancer stem cell traits, which are closely linked to tumor invasion and poor survival outcomes [35,53].

We identified three distinct CAF subtypes—myCAFs, csCAFs, and apCAFs—consistent with previous reports [9,10]. This study specifically focused on myCAFs, which are characterized by elevated ECM remodeling activity and a pronounced capacity to be associated with EMT in tumor cells. These cells actively participate in tumor–stromal interactions by secreting THBS2 and FN1. Notably, FN1 promotes tumor metastasis by binding to membrane receptors and activating the FAK/src-ERK and AKT signaling pathways [54,55], suggesting that myCAFs may contribute to PDAC progression and metastasis. Our analysis further indicated that csCAFs exhibit functional features associated with immune regulation and inflammatory responses and express iCAF activation markers, suggesting a potential transition towards an iCAF-like state [41]. CAFs originate from multiple sources, including stellate cells, bone marrow-derived mesenchymal stem cells, and endothelial, mesothelial, and epithelial cells undergoing EMT [12,14,56]. Consistent with this, our findings suggested that myCAFs in PDAC may originate from two distinct sources: acquisition of myCAF-like features by Ductal-T0 cells through EMT-associated transcriptional state transitions and, at least in part, from PSCs.

This study is the first to describe the cellular sources of FN1 and THBS2 in PDAC, showing that these proteins are primarily secreted by myCAFs. This finding extends the current understanding that CAFs are associated with PDAC progression through FN1/THBS2-mediated integrin pathways and that TGF-β1 upregulates *THBS2* expression, offering a cellular-origin perspective on CAF–tumor cell interactions [17,57]. In lung adenocarcinoma, *THBS2*^+^ myCAFs exhibit notable spatial colocalization with tumor cells; THBS2 secreted by these cells engages the SDC4 receptor on tumor cells, thereby activating EMT signaling [16]. CXCL12 is predominantly produced by csCAFs and binds to CXCR4 on T cells, B cells, and macrophages. Previous studies have generally regarded iCAFs and myCAFs as the main sources of *CXCL12* [58,59]. Although iCAFs and csCAFs share certain features, they are distinct subpopulations [60].

THBS2 secreted by myCAFs is predicted to interact with CD47 on tumor cells, facilitating tumor progression via MAPK/ERK5 pathway activation. *THBS2* may be functionally implicated in the processes of CAF activation, EMT, and chemotherapy resistance [43,61]. Further analysis revealed that *THBS2* knockdown upregulated the epithelial marker E-cadherin and downregulated the mesenchymal marker vimentin (*VIM*) [62,63,64], suggesting that *THBS2* may promote cancer cell migration by inducing EMT. *THBS2* is a key regulator of the immune exclusion phenotype [65]. Consistent with the findings of Wu and Pei regarding ECM-remodeling fibroblasts and EMT-related epithelial cells [52,59], myCAFs characterized by EMT and ECM activation are predominantly concentrated at the tumor front, where ECM organization takes place, and they may contribute to reduced immune presence within the tumor core.

SDC1, SDC4, and ITGA3 are key molecules that are associated with interactions with the ECM and are integral to EMT. This study identified that malignant ductal cells express elevated levels of *SDC1*, *SDC4*, and *ITGA3* [66], with the Ductal-T0 subpopulation showing the most pronounced upregulation, indicative of high basal/EMT activity. Pseudotime analysis placed these cells at the terminal branch of the invasion trajectory, suggesting their potential role in driving the epithelial-to-mesenchymal phenotypic transition, consistent with the findings of Xie et al. [67]. ITGA3, through its interactions with other ECM receptors, may promote tumor cell proliferation, invasion, and migration by activating the PI3K-Akt signaling pathway [27,68]. Inhibiting *SDC1* expression impairs EMT in pancreatic cancer cell lines [69], whereas *SDC4* positively regulates TGF-β1-induced EMT in cancer cells via *SNAI1* [24]. Multiple studies have reported that silencing or inhibiting *SDC1*, *SDC4*, or *ITGA3* markedly reduces cancer cell growth and metastasis [24,26,69,70], suggesting that these molecules represent promising candidates for future therapeutic investigation.

This study had several limitations. First, all findings are derived from computational analyses of publicly available retrospective datasets (TCGA, GEO, CPTAC, and GSA), which precludes causal inference and introduces potential selection bias. Moreover, inter-patient heterogeneity (treatment history, genetic background, disease stage) may confound certain results. Second, trajectory inference methods (pseudotime, scTour, CellRank) predict transcriptional continuity based on transcriptomic similarity, but cannot establish definitive lineage relationships without lineage-tracing studies. Third, ST data are limited to eight sections from three datasets, which may not fully represent PDAC heterogeneity across stages and anatomical sites. Finally, the proposed THBS2/FN1-SDC/Integrin axis and the inferred Ductal-T0-to-myCAF transition remain hypotheses requiring experimental verification, and the specific signaling pathways linking ECM–receptor interactions to EMT activation warrant further mechanistic investigation. To address these gaps, future work will employ gene knockdown of key candidates, in vitro co-culture systems, and ligand-blocking experiments using anti-THBS2 and anti-FN1 neutralizing antibodies to provide functional and mechanistic validation.

In conclusion, we described a malignant ductal cell subpopulation, termed Ductal-T0, that showed transcriptional features associated with phenotypic plasticity. As a key component of the EMT niche, myCAFs secrete THBS2 and FN1, which are predicted to engage the ITGA3/ITGB1/SDC1/SDC4 receptor axes in Ductal-T0 cells. This interaction may contribute to the EMT program and is associated with the acquisition of myCAF-like features, potentially enhancing tumor cell invasiveness. Notably, *SDC1*, *SDC4*, and *ITGA3* are associated with the epithelial-to-mesenchymal phenotypic transition and represent promising candidates for future therapeutic investigation in PDAC metastasis. Additionally, ECM deposition and remodeling at the tumor margin may contribute to the establishment of an immune exclusion zone.

## 4. Materials and Methods

### 4.1. Data Collection

The single-cell RNA-seq dataset CRA001160 was downloaded from the Genome Sequence Archive (GSA) of the National Genomics Data Center (https://ngdc.cncb.ac.cn/gsa/ (accessed on 3 March 2025)) [71], which included 24 primary tumor samples and 11 normal samples. Three spatial transcriptomic (ST) datasets of PDAC were acquired from the Gene Expression Omnibus (GSE211895 [72], GSE274557 [59], and GSE274673 [73]), and a total of eight slices were utilized for further analysis (Supplementary Tables S4 and S5).

The preprocessed proteomic dataset OEP00004100 was collected from the National Omics Data Encyclopedia (https://www.biosino.org/node/home (accessed on 22 October 2025)), which included 281 samples (191 treatment-naïve tumors and 90 paired tumor-adjacent tissues) (Supplementary Table S6) [74]. We also downloaded mRNA and proteomic expression datasets from the CPTAC-PDAC project from the LinkedOmics database (https://www.linkedomics.org/data_download/CPTAC-PDAC/ (accessed on 22 October 2025)) with 211 samples (135 PDACs, 67 paired normal-adjacent tissues, and 9 normal pancreatic duct tissues) (Supplementary Table S7) [75].

### 4.2. scRNA-Seq Data Processing and Cell Annotation

All data processing analyses were conducted using the R platform (v4.4.1) and the Seurat (v5.1.0) package. Low-quality cells were filtered using the following criteria: (1) a minimum of 200 detected genes and (2) a mitochondrial gene percentage exceeding 10%. After normalization and log transformation, the top 2000 highly variable genes were selected using FindVariableFeatures function. To reduce data dimensionality, principal components (PCs) were calculated based on highly variable genes, and we finally selected 48 as the appropriate number of PCs after several rounds of analysis and comparison. All samples were integrated using Harmony (v1.2.0) to remove batch effects. Clustering was performed using the FindNeighbors and FindClusters functions with resolution = 1.2, and clusters were visualized using uniform manifold approximation and projection (UMAP). The FindAllMarkers function was used to identify differentially expressed genes (DEGs) across clusters, with parameters set to ‘min.pct = 0.25’, and ‘logfc.threshold = 0.25’. Upregulated DEGs were set with ‘pct.1 > 0.1’, ‘*p*_val < 0.05’, and ‘avg_log2FC > 0.5’.

Cell annotation was conducted manually, and we collected cell markers of pancreatic tissue from previous studies: (1) for T cells, the markers used were *CD3D*, *CD3E*, *CD3G*, and *CD8A*; (2) for B cells, the markers were *MS4A1*, *CD79A*, and *CD79B*; (3) macrophages were identified using markers *AIF1*, *CD14*, and *CD68*; (4) endothelial cells were characterized by the markers *CDH5*, *CLDN5*, and *ENG*; (5) *PRSS1*, *CTRB1*, *CTRB2*, and *REG1B* for acinar cells; (6) *CHGA*, *GHGB*, and *IAPP* for endocrine cells; (7) *ACTA2*, *PDGFRB*, and *RGS5* for stellate cells; (8) the marker genes for fibroblasts were *COL1A1*, *DCN*, and *COL6A1*; (9) the epithelial ductal cells were clearly identified as two types, ductal-1 (*AMBP*, *CFTR*, and *MMP7*) and ductal-2 (malignant epithelial cell, *KRT7*, *KRT19*, *SLPI*, and *TSPAN8*).

This workflow was the first clustering analysis that we identified major cell types, the second enabled us to have a comprehensive overview of specific individual subtypes in ductal-2 and fibroblast cells.

### 4.3. Spatial Transcriptomic Data Analysis

For ST data, we analyzed the gene–spot matrices generated from processed Visium and Xenium samples using the Seurat (v5.1.0) package. Normalization of each spot was performed using SCTransform function and the top 30 principal components (PCs) were selected via RunPCA function for dimensionality reduction. We applied FindNeighbors and FindClusters functions (with a resolution of 0.8 for Visium and 0.3 for Xenium) to generate distinct ST spot clusters, visualized by RunUMAP function. Based on identifying “similar spot pairs” (also referred to as anchors) between scRNA-seq and Visium data, TransferData function was used to project cell type labels from scRNA-seq data onto the ST data. Given that each spot contains multiple cells, we subsequently integrated scRNA-seq data using SPOTlight (v1.5.1) for the deconvolution of ST capture locations to predict the dominant cell types and ratios [76]. Spatial feature expression maps were generated using the SpatialFeaturePlot function to visualize marker expression patterns in spatial contexts. For the Xenium data, cell type deconvolution was performed using the RCTD method to predict the cell types present at each spot in the Xenium dataset by leveraging cell type annotations from scRNA-seq datasets [77].

Based on the Squidpy (v1.9.6) Python library [78], we defined neighboring spots as those within one hexagonal ring around each spot using sq.gr.spatial_neighbors function and constructed a cell neighborhood matrix. Subsequently, the interaction matrices features were calculated using sq.gr.interaction_matrix function.

### 4.4. InferCNV Analysis

To elucidate the characteristics of epithelial ductal cells, we utilized the InferCNV (v1.20.0) package (https://github.com/broadinstitute/inferCNV (accessed on 25 February 2025)) to calculate the copy number variation (CNV) scores for epithelial ductal cells (ductal-1 and ductal-2 cell clusters). The T cells served as a normal reference, with a cutoff of 0.1 and denoising enabled. CNV heatmaps (hclust_method = “ward.D2”) and violin plots of CNV scores for epithelial ductal cell clusters were generated.

### 4.5. BayesPrism Deconvolution Analysis

BayesPrism (v2.2.0) was employed for cell composition deconvolution of bulk transcriptomic data from 147 TCGA-PAAD tumor samples [79]. The single-cell transcriptomic dataset generated in this study served as the reference expression matrix, with cell subpopulations containing fewer than 200 cells (apCAFs and Ductal-T5) being excluded. The relative proportions of distinct cell subpopulations in the samples were calculated. Following the removal of ribosomal protein genes and mitochondrial genes, 16,011 protein-coding genes were retained for constructing the ‘new.prism’ object (with ‘key = “Ductal 2”’, designating ductal-2 as the malignant cell population; all other parameters were set to default), from which the compositional proportions of various cell subpopulations were derived. Survival analysis was performed using the survival (v3.8-3) package. Based on the compositional proportion of the ductal-2 subpopulation in each sample, the optimal cutoff value was determined using the surv_cutpoint function from the survminer (v0.5.0) package to stratify the samples into high- and low-proportion groups.

### 4.6. Pseudotime Analysis/Trajectory Analysis

Monocle2 (v2.32.0) was used to construct the differentiation trajectories of (1) ductal-2 cells, (2) fibroblasts and stellate cells, and (3) myCAFs and ductal-2 cells, aiming to clarify the cellular differentiation trajectories [80].

Highly variable genes were selected as those with mean expression ≥ 0.1 and empirical dispersion ≥ fitted dispersion value. Dimensionality reduction was performed using the DDRTree method. Cells and genes were visualized using the plot_cell_trajectory and plot_genes_in_pseudotime functions to demonstrate cell differentiation trajectories and gene expression changes over pseudotime, respectively. The Branched expression analysis modeling (BEAM) method identified genes that distinguished different cell branches, and EnrichCluster function was used to perform The Kyoto Encyclopedia of Genes and Genomes (KEGG) pathway enrichment analysis on the top 100 differentially expressed genes that were significantly dependent on branches.

To further investigate the integration of myCAF and ductal cell 2, principal components PC3 to PC14 were selected for subsequent dimensionality reduction and clustering, followed by scTour (v1.0.0) and CellRank2 (v2.0.7) analyses [81,82]. CellRank2 was used to compute the transition matrix via the PseudotimeKernel based on Monocle2 pseudotime, with Ductal-T1 designated as the starting point. To balance the directionality of pseudotime and transcriptional similarity, the PseudotimeKernel was combined with the similarity-based ConnectivityKernel, with weights of 0.8 and 0.2, respectively. The GPCCA estimator was employed to identify the initial and terminal states, and compute_fate_probabilities functions was used to calculate the probability of each cell reaching each terminal state. scTour is a deep generative model-based method for inferring single-cell dynamics. During model training, the default parameters of sct.train.Trainer function was employed. The trained model was applied to infer the developmental pseudotime, and both pseudotime and vector fields were visualized on the UMAP plot.

### 4.7. Cell–Cell Communications Analysis

Ligand–receptor interactions were inferred using CellChat (v1.6.1) [83]. We utilized the createCellChat function to import gene expression data, selecting the human database for intercellular communication analysis, which covered secretion signaling, cell-to-cell contact, and ECM receptor modules. The identifyOverExpressedGenes function identifies significantly overexpressed ligand–receptor genes across cell types, whereas the identifyOverExpressedInteractions function detects highly expressed ligand–receptor pairs between cell type pairs. The computeCommunProb function calculates the intercellular communication probabilities. The netVisual_heatmap function visualizes cell interaction networks, and the netAnalysis/signalingRole_heatmap functions highlight the signaling roles of cell subpopulations and key contributing signals.

NicheNet R package (v2.20) was used to infer the interaction between myCAF and Ductal-T0 [84]. The criteria for identifying receptors and ligands using the get_expressed_genes function were as follows: the receptor must be expressed in at least 25% of Ductal-T0 cells, and the ligand must be expressed in at least 50% of myCAF cells. The pEMT gene set (https://zenodo.org/record/3260758/files/pemt_signature.txt (accessed on 8 July 2025)) was used as the target gene set to study how CAFs induce EMT in malignant cells. Ligand activity analysis was performed using predict_ligand_activities functions, and the top 30 ligands ranked by the area under the precision–recall curve (AUPR) were used to construct an AUPR heatmap.

The prior knowledge network of NicheNet (data source: https://zenodo.org/record/3260758 (accessed on 8 July 2025)) was used to predict the regulatory pathways from ligands to target genes. Based on the results of CellChat and NicheNet, FN1 and THBS2 were considered as potential ligands, and their putative receptors (ITGB1, ITGA3, SDC1, SDC4) along with the corresponding target genes were integrated to predict the regulatory pathways from signaling intermediates to transcription factors. The 20 transcription factors (TFs) with the highest interaction weights with target genes were selected as core regulators for subsequent analysis. By calculating the distances between receptors and transcription factors in the signaling network, the ligand–target gene signaling pathways were ultimately obtained (see the method at: https://workflows.omnipathdb.org/nichenet1.html (accessed on 30 April 2026)).

### 4.8. High-Dimensional Weighted Gene Co-Expression Network Analysis

High-dimensional weighted gene co-expression network analysis (hdWGCNA, v0.4.11) is an R package developed for scRNA-seq to identify disease-relevant co-expression network modules [85,86]. The default parameters of SetDataExpr function were used to construct an expression matrix and generate the corresponding MES data objects. The TestSoftPowers function calculated the soft power threshold (threshold = 8). We then used the default parameters of the ConstructNetwork function to construct a gene co-expression network and identified ten gene modules. The ModuleConnectivity function calculates the degree of connectivity (kME) within the module, and the ModuleExprScore function is combined with the UCell algorithm to calculate the central gene signature score for each module.

To decipher the co-expression relationship between *THBS2* and module genes and to identify gene modules significantly associated with the expression pattern of *THBS2*, 10,114 protein-coding genes retained from the hdWGCNA analysis were used. The average expression of *THBS2* in myCAF across samples was used as the reference, with Ductal-T0 serving as the control. The Spearman correlation was then calculated between the average expression of *THBS2* and the average expression of genes within each module across the same samples. Furthermore, the EMT-related gene sets (Hallmark EMT, pEMT, and the pEMT signature derived from NicheNet) were used to evaluate the enrichment significance of each module for the EMT gene sets via Fisher’s exact test, with 10,114 protein-coding genes as the background.

### 4.9. Functional Enrichment Analysis

Differential expression analysis was performed using the limma R package (v3.62.2) with the lmFit, eBayes, and topTable functions [87]. Significantly differentially expressed mRNAs and proteins were identified based on the criteria of |log2FC| > 1.0 and adjusted *p*-value < 0.05.

KEGG pathway and Gene Ontology (GO) functional enrichment analyses were conducted using the clusterProfiler R package (v4.12.0) [88]. Significantly enriched terms were defined using a threshold of BH-adjusted *p*-value < 0.05. Gene set enrichment analysis (GSEA) was performed using the GSEA function of the clusterProfiler package. Genes were pre-ranked in descending order based on avg_log2FC prior to pathway scoring. Statistical significance was set at a BH-adjusted *p*-value < 0.05.

### 4.10. Survival Analysis

Based on the optimal cutoff values calculated by the surv_cutpoint function from the Survminer package (v0.5.0), mRNA and protein expression levels were dichotomized into high- and low-expression groups, respectively. Survival curves were subsequently plotted using the surv_group function from the IOBR R package (v0.99.99) [89].

### 4.11. Gene Set Scoring

Gene set enrichment scores for scRNA-seq data were computed using the single-sample gene set enrichment analysis (ssGSEA) method from the GSVA R package (v1.38.2) [90]. Gene sets for the EMT Hallmark pathway were obtained from the Molecular Signatures Database (MSigDB; https://www.gsea-msigdb.org/gsea/msigdb (accessed on 27 May 2025)) via the msigdbr R package [91]. EMT-associated signatures were derived from previously published studies (Supplementary Tables S8 and S9) [35,42,92,93]. The gene sets were scored using the AddModuleScore function to evaluate their expression activity.

### 4.12. scRANK Analysis of Drug-Responsive Cell Types in Malignant Ductal Cells

We applied the scRank R package (v1.0) to predict drug response grades across pancreatic malignant ductal cell subtypes for *SDC1* [94]. For the target, we constructed an antagonist perturbation model using the CreateScRank function, built a gene regulatory network for targeted perturbation with the Constr_net function (default parameters), and ranked cell types by predicted sensitivity using the rank_celltype function.

### 4.13. Statistical Analysis

All statistical analyses were performed in R (v4.1.0). For two-group comparisons, the unpaired Student’s *t*-test and the Wilcoxon rank-sum test were used. For multi-group comparisons, the Kruskal–Wallis test was applied. Correlation analyses were performed using both Pearson’s and Spearman’s correlation coefficients. Unless otherwise specified, all tests were two-sided, and *p*-value < 0.05 was considered statistically significant, multiple-testing correction was performed using the Benjamini–Hochberg method where applicable, and adjusted *p*-value < 0.05. Detailed statistical information, including sample sizes, specific tests used, and correction methods, is also provided in the corresponding figures and their legends.

## Data Availability

All datasets presented in this study can be downloaded from online websites. Further inquiries can be directed to the corresponding author Xi Liu at liuxi@gdpu.edu.cn.

## Supplementary Materials

The following supporting information can be downloaded at https://www.mdpi.com/article/10.3390/ijms27156951/s1.

## Abbreviations

apCAFs	Antigen-presenting cancer-associated fibroblasts
BEAM	Branched expression analysis modeling
CAFs	Cancer-associated fibroblasts
CNV	Copy number variation
CPTAC	Clinical Proteomic Tumor Analysis Consortium
csCAFs	Complement-secreting cancer-associated fibroblasts
DEGs	Differentially expressed genes
ECM	Extracellular matrix
EMT	Epithelial–mesenchymal transition
GEO	Gene Expression Omnibus
GO	Gene Ontology
GPCCA	Generalized Perron cluster analysis
GSA	Genome Sequence Archive
GSEA	Gene set enrichment analysis
hdWGCNA	High-dimensional weighted gene co-expression network analysis
HIF-1α	Hypoxia-inducible factor 1-alpha
HPA	Human Protein Atlas
iCAFs	Inflammatory cancer-associated fibroblasts
KEGG	Kyoto Encyclopedia of Genes and Genomes
kME	Module eigengene connectivity
MES	Mesenchymal
MSigDB	Molecular Signatures Database
myCAFs	Myofibroblastic cancer-associated fibroblasts
NEN	Neuroendocrine-like
PAAD	Pancreatic adenocarcinoma
PCs	Principal components
PDAC	Pancreatic ductal adenocarcinoma
pEMT	Partial epithelial–mesenchymal transition
PSCs	Pancreatic stellate cells
qPSCs	Quiescent pancreatic stellate cells
RCTD	Robust cell type decomposition
scRNA-seq	Single-cell RNA sequencing
smPSCs	Smooth muscle pancreatic stellate cells
ssGSEA	Single-sample gene set enrichment analysis
ST	Spatial transcriptomics/Spatial transcriptomic
TCGA	The Cancer Genome Atlas
TFs	Transcription factors
TME	Tumor microenvironment
UMAP	Uniform manifold approximation and projection
