# Supplementary material for: Myofibroblastic CAF and Malignant Ductal Cell Crosstalk Drives Epithelial–Mesenchymal Transition and Progression in Pancreatic Ductal Adenocarcinoma via THBS2-SDC/Integrin Axes
Source: Int J Mol Sci. 2026 Aug 2;27(15):6951. doi: 10.3390/ijms27156951 (PMC13466557; doi:10.3390/ijms27156951)
Supplement: Supplementary file 1 [file ijms-27-06951-s001.zip › Supplementary Figures-revised-260801.pdf]

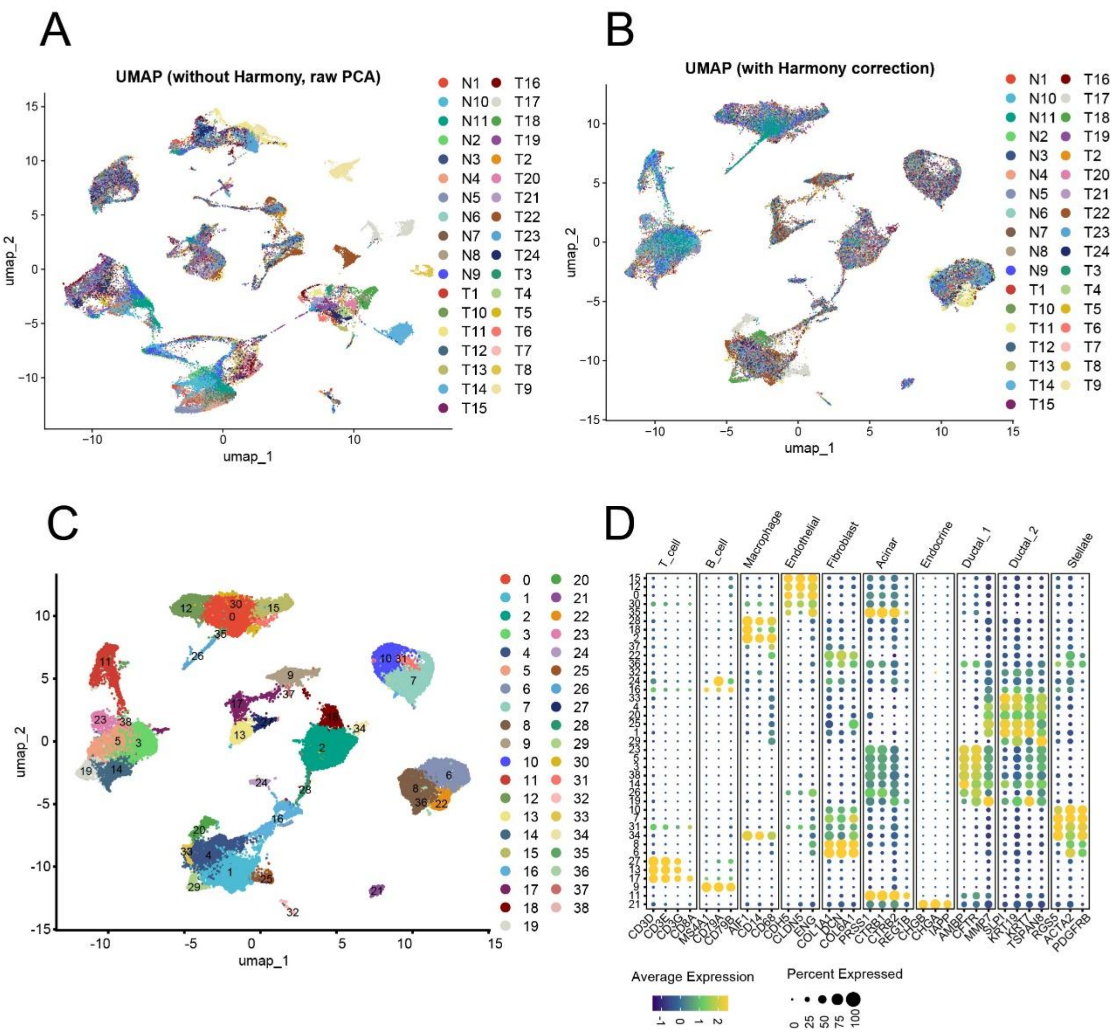

Supplementary Figure S1. Construction of Single-Cell Transcriptomic Atlas of PDAC. (A) Before Harmony integration, colored by sample source, showing clear batch segregation. (B) After Harmony integration, colored by sample origin, demonstrating successful batch mixing and distinct cell-type clustering. (C) After Harmony integration, colored by 39 major cell clusters. (D) Dotplot showing average expression of marker genes across cell clusters.

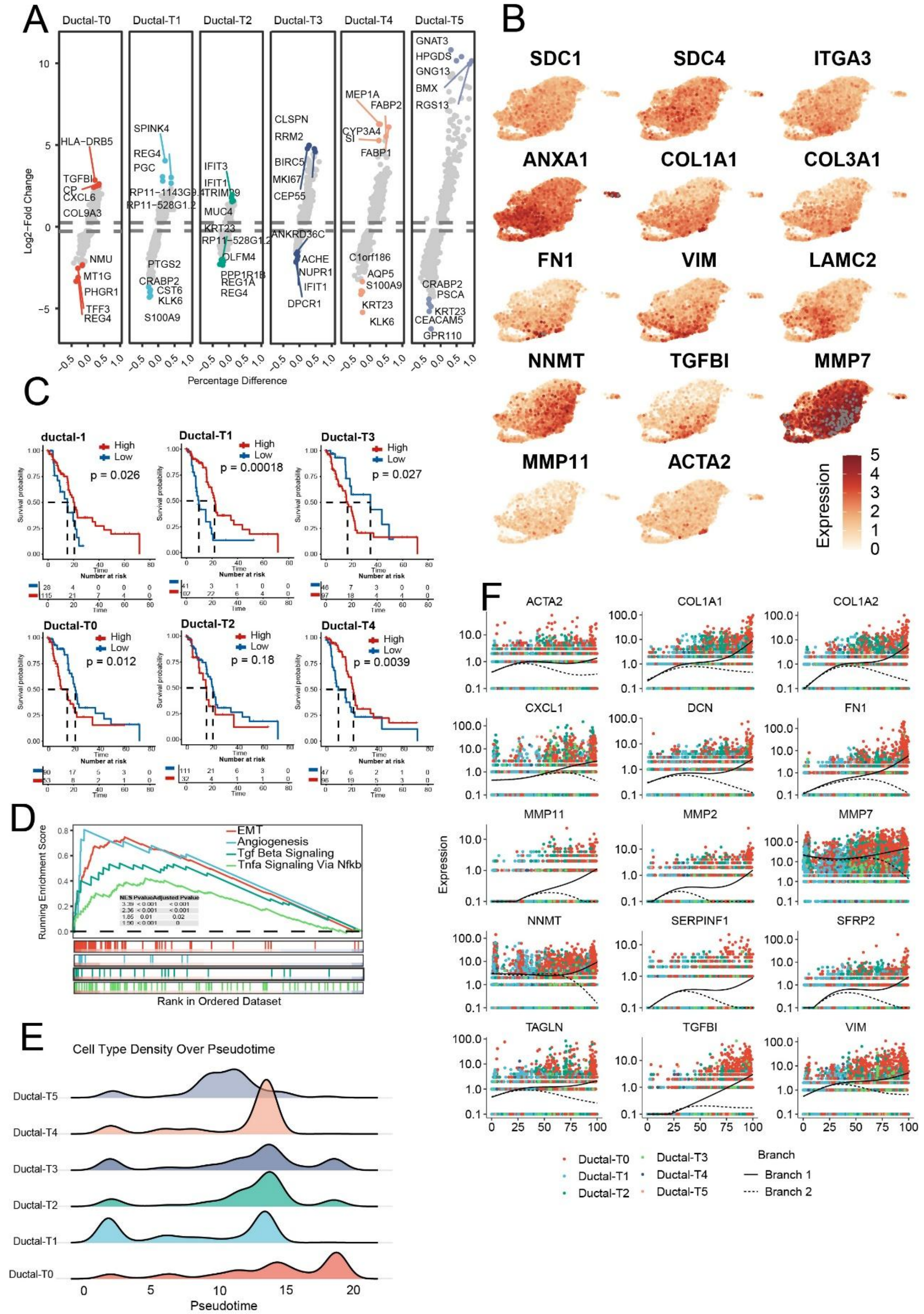
A
Ductal-T0
Ductal-T1
Ductal-T2
Ductal-T3
Ductal-T4
Ductal-T5
Log2-Fold Change
Percentage Difference
HLA-DRB5
TGFBI
CP
CXCL6
COL9A3
NMU
MT1G
PHGR1
TFF3
REG4
SPINK4
REG4
PGC
RP11-1143G9.4
RP11-528G1.2
PTGS2
CRABP2
CST6
KLK6
S100A9
IFIT3
IFIT1
TRIM29
MUC4
KRT23
RP11-528G1.2
OLFM4
PPP1R1B
REG1A
REG4
CLSPN
RRM2
BIRC5
MKI67
CEP55
ANKRD36C
ACHE
NUPR1
IFIT1
DPCR1
MEP1A
FABP2
CYP3A4
SI
FABP1
C1orf186
AQP5
S100A9
KRT23
KLK6
GNAT3
HPGDS
GNG13
BMX
RGS13
CRABP2
PSCA
KRT23
CEACAM5
GPR110
B
SDC1
SDC4
ITGA3
ANXA1
COL1A1
COL3A1
FN1
VIM
LAMC2
NNMT
TGFBI
MMP7
MMP11
ACTA2
Expression
C
ductal-1
Ductal-T1
Ductal-T3
Ductal-T0
Ductal-T2
Ductal-T4
High
Low
p = 0.026
p = 0.00018
p = 0.027
p = 0.012
p = 0.18
p = 0.0039
Survival probability
Time
Number at risk
D
EMT
Angiogenesis
Tgf Beta Signaling
Tnfa Signaling Via Nfkb
Running Enrichment Score
Rank in Ordered Dataset
E
Cell Type Density Over Pseudotime
Ductal-T5
Ductal-T4
Ductal-T3
Ductal-T2
Ductal-T1
Ductal-T0
Pseudotime
F
ACTA2
COL1A1
COL1A2
CXCL1
DCN
FN1
MMP11
MMP2
MMP7
NNMT
SERPINF1
SFRP2
TAGLN
TGFBI
VIM
Expression
Ductal-T0
Ductal-T1
Ductal-T2
Ductal-T3
Ductal-T4
Ductal-T5
Branch
Branch 1
Branch 2

Supplementary Figure S2. Heterogeneity and differentiation trajectory of malignant ductal cell subpopulations. (A) Volcano plot showing differentially expressed genes among malignant ductal cell subpopulations (two-sided Wilcoxon rank-sum test, Seurat FindAllMarkers, default parameters). (B) UMAP plot showing expression distribution of EMT-related genes. (C) Kaplan-Meier curves showing prognostic differences among patient groups stratified by celltype abundance estimated by deconvolution analysis of TCGA-PAAD data (log-rank test). (D) GSEA showing Hallmark pathways upregulated in Ductal-T0 cells (Permutation-based significance with Benjamini–Hochberg FDR correction). (E) Cell density distribution of malignant ductal cell subpopulations along the pseudotime axis. (F) Expression trends of EMT-related genes along pseudotime.

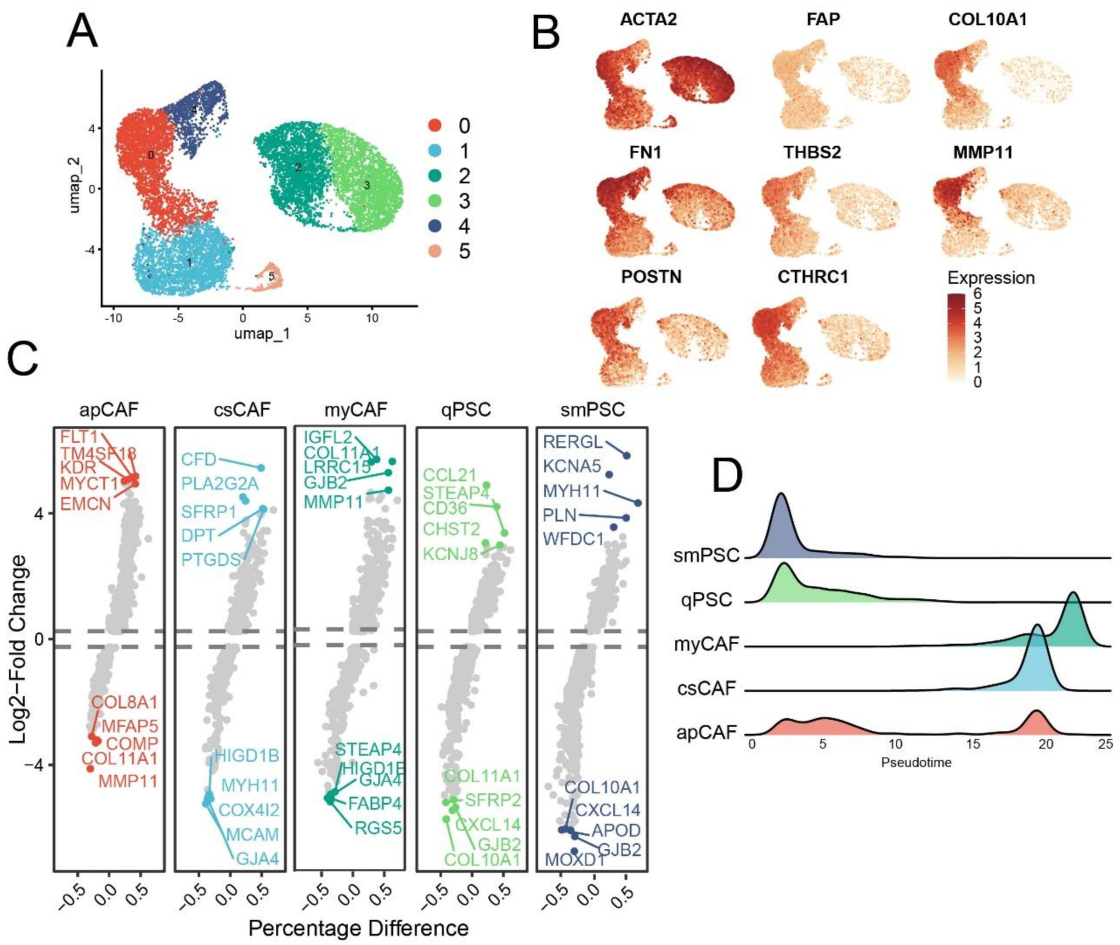

Supplementary Figure S3. Heterogeneity and characterization of CAFs subpopulations. (A) UMAP plot showing subclustering results, colored by distinct cell clusters. (B) UMAP plot showing expression distribution of specific marker genes in myCAFs. (C) Volcano plot showing differentially expressed genes among CAFs subpopulations (two-sided Wilcoxon rank-sum test, Seurat FindAllMarkers, default parameters). (D) Cell density distribution of cell subpopulations.

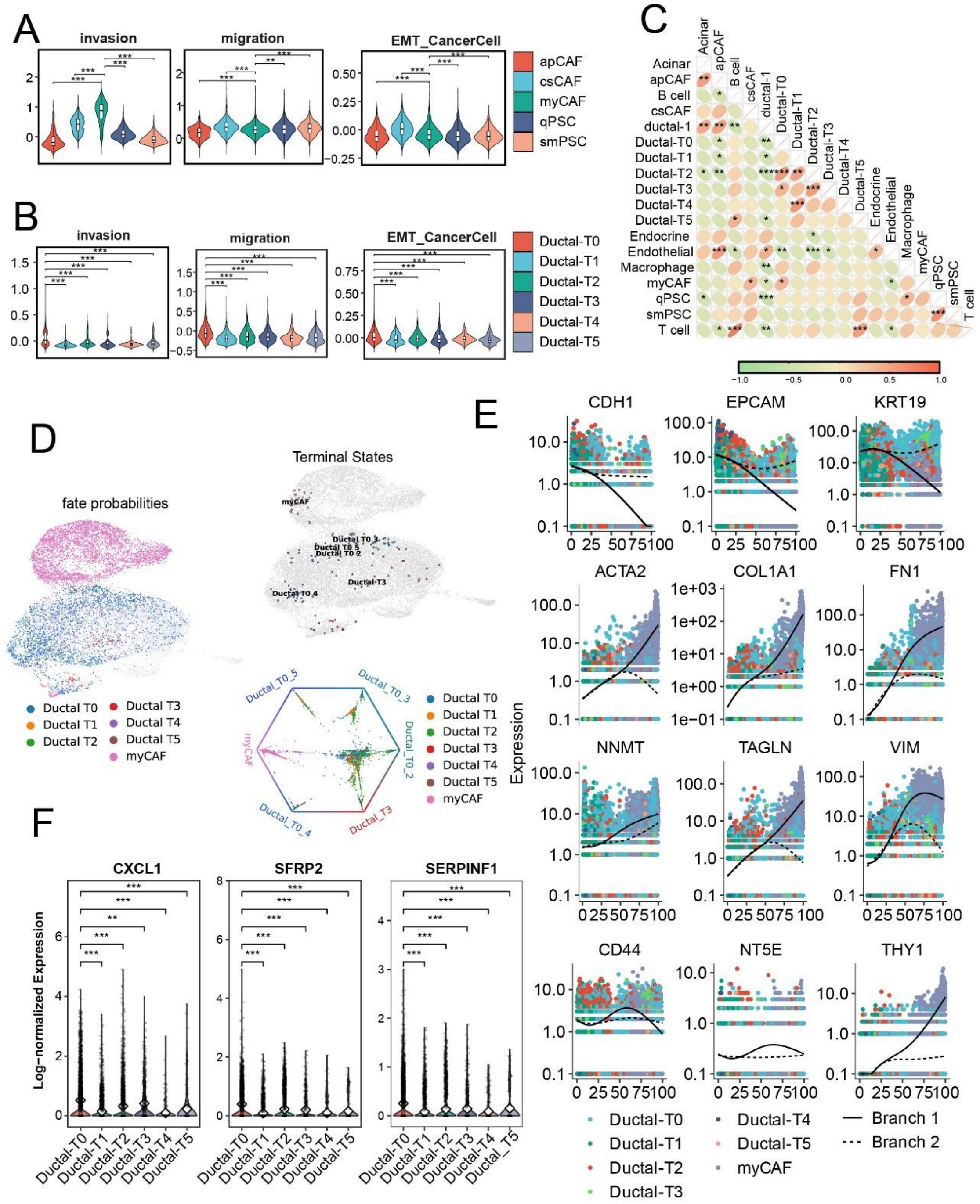

A
invasion
migration
EMT_CancerCell
apCAF
csCAF
myCAF
qPSC
smPSC
B
Ductal-T0
Ductal-T1
Ductal-T2
Ductal-T3
Ductal-T4
Ductal-T5
C
Acinar
apCAF
B cell
csCAF
ductal-1
Endocrine
Endothelial
Macrophage
qPSC
smPSC
T cell
D
fate probabilities
Terminal States
Ductal T0
Ductal T1
Ductal T2
Ductal T3
Ductal T4
Ductal T5
myCAF
E
CDH1
EPCAM
KRT19
ACTA2
COL1A1
FN1
NNMT
TAGLN
VIM
CD44
NT5E
THY1
Expression
Branch 1
Branch 2
F
CXCL1
SFRP2
SERPINF1
Log-normalized Expression

Supplementary Figure S4. EMT signature characterization across CAFs and malignant ductal cell subpopulations. (A) Violin plots showing the distribution of invasion, migration-related gene set scores and EMT-cancer cell gene set scores, as defined by Wei et. al., in myCAFs. (B) Violin plots showing the distribution of invasion, migration-related gene set scores and EMT-cancer cell gene set scores, as defined by Wei et al., in malignant ductal cell subpopulations. (C) Correlation heatmap of cell type abundances in single-cell data. Color intensity indicates correlation strength. (D) CellRank2 trajectory analysis. Six terminal states defined along pseudotime, the fate probabilities of cells toward each terminal state, and a circular projection based on these probabilities are shown. (E) Expression dynamics of epithelial markers, EMT signature genes, and stemness related genes along pseudotime. (F) Violin plots showing expression levels of CAFs markers (*CXCL1*, *SFRP2*, and *SERPINF1*) associated with EMT in malignant ductal cell subpopulations. Statistical information: All tests were two-sided. Panels A–B, and F: pairwise Wilcoxon rank-sum tests. Panel C: two-sided Pearson's correlation coefficient test. **$p < 0.01$, ***$p < 0.001$.

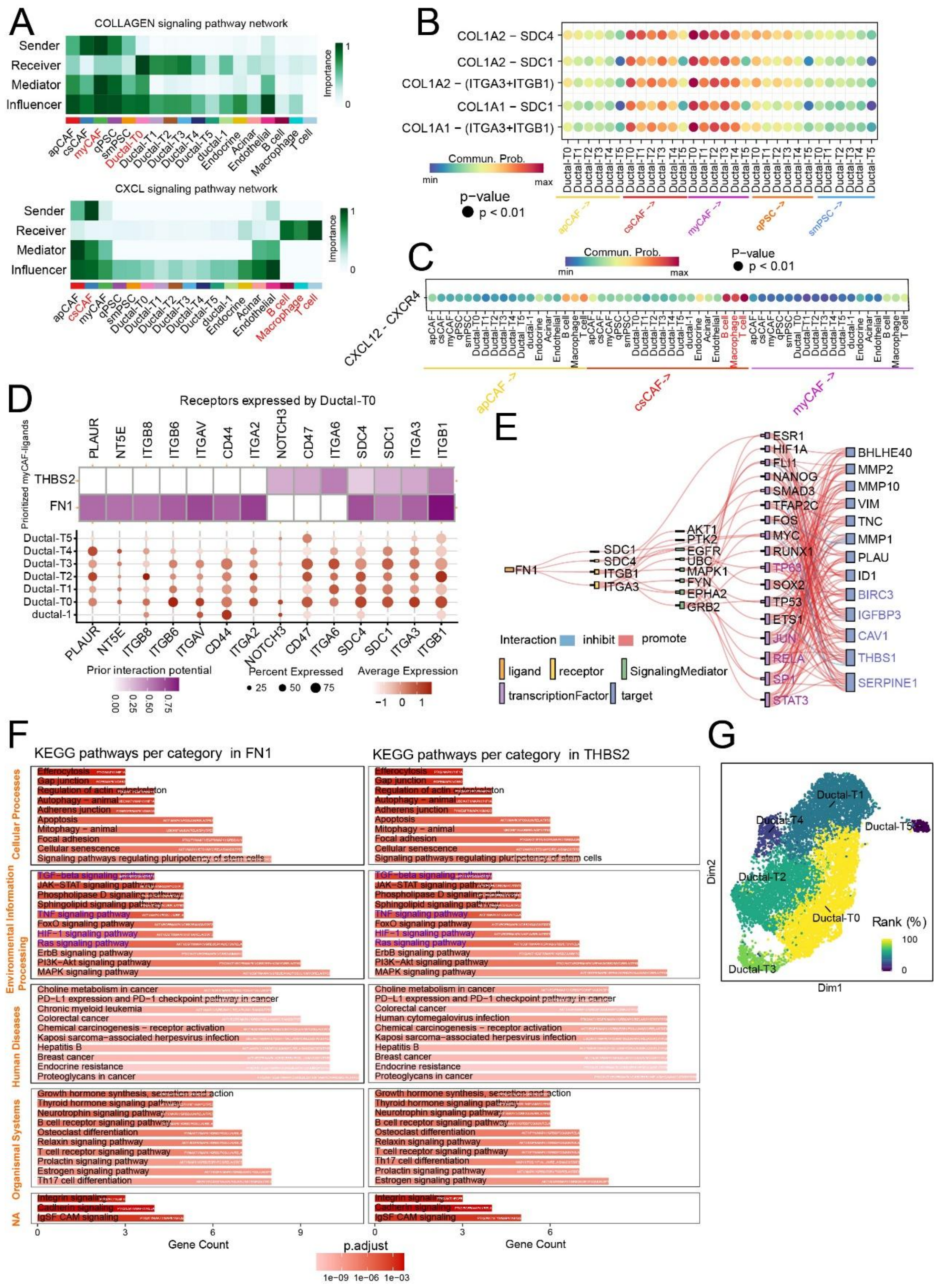
A
COLLAGEN signaling pathway network
Sender
Receiver
Mediator
Influencer
Importance
CXCL signaling pathway network
B
COL1A2 − SDC4
COL1A2 − SDC1
COL1A2 − (ITGA3+ITGB1)
COL1A1 − SDC1
COL1A1 − (ITGA3+ITGB1)
Commun. Prob.
p−value
p < 0.01
apCAF ->
csCAF ->
myCAF ->
qPSC ->
smPSC ->
C
CXCL12 - CXCR4
D
Receptors expressed by Ductal-T0
Prioritized myCAF-ligands
THBS2
FN1
Prior interaction potential
Percent Expressed
Average Expression
E
Interaction
inhibit
promote
ligand
receptor
SignalingMediator
transcriptionFactor
target
F
KEGG pathways per category in FN1
KEGG pathways per category in THBS2
Cellular Processes
Environmental Information Processing
Human Diseases
Organismal Systems
NA
Gene Count
p.adjust
G
Rank (%)
Dim1
Dim2

Supplementary Figure S5. FN1/THBS2-mediated communication between myCAFs and Ductal-T0 cells. (A) Network diagram of the COLLAGEN/CXCL signaling pathway. (B) Bubble plot showing communication probabilities of key ligand-receptor pairs in the COLLAGEN signaling pathway across different cell types. (C) Bubble plot showing communication probabilities of key ligand-receptor pairs in the CXCL signaling pathway between csCAFs and immune cells (CellChat-inferred communication probability assessed by permutation test). (D) NicheNet prediction of *THBS2* and *FN1* as key ligands binding to corresponding receptors. (E) Network diagram of potential signaling pathways through which *FN1* regulates target genes. Colored fonts indicate the top 10 nodes calculated by the MCC algorithm in Cytoscape. (F) Intermediate nodes in the *FN1* and *THBS2* regulatory network and their KEGG pathway enrichment analysis results (One-tailed hypergeometric test with Benjamini–Hochberg FDR correction). (G) Prediction of *SDC1* antagonist response in malignant ductal cell subtypes using scRANK.

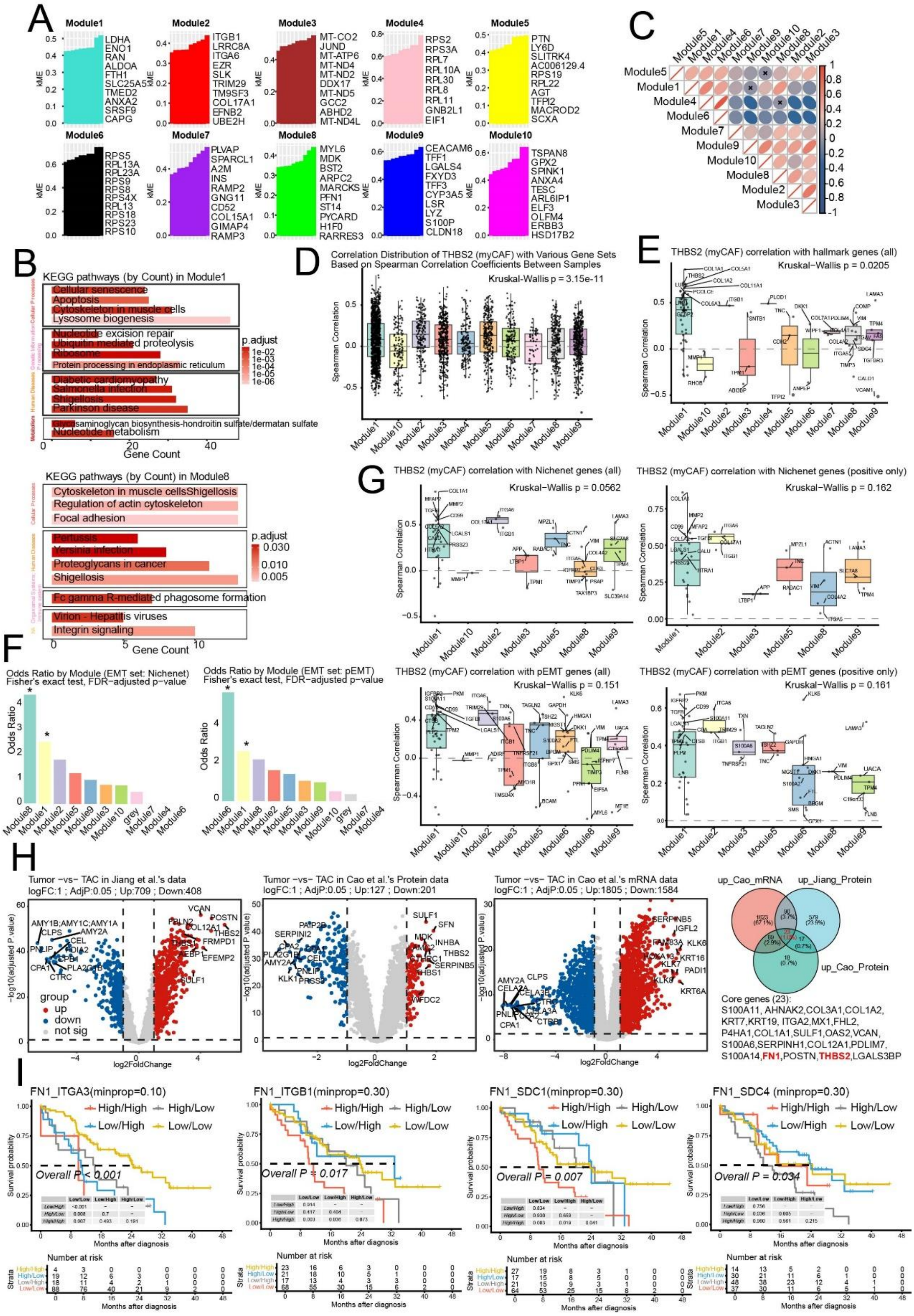
A
Module1
LDHA
ENO1
RAN
ALDOA
FTH1
SLC25A5
ANXA2
TMED2
RPS8
SRSF9
CAPG
Module2
ITGB1
LRRC8A
ITGA6
EZR
SLK
TRIM29
TM9SF3
COL17A1
EFNB2
UBE2H
Module3
MT-CO2
JUND
MT-ATP6
MT-ND4
MT-ND2
DDX17
MT-ND5
GCC2
ABHD2
MT-ND4L
Module4
RPS2
RPS3A
RPL7
RPL10A
RPL30
RPL8
RPL11
GNB2L1
EIF1
Module5
PTN
LY6D
SLITRK4
AC006129.4
RPS19
RPL22
AGT
TFPI2
MACROD2
SCXA
Module6
RPS5
RPL13A
RPL23A
RPS9
RPS8
RPS4X
RPL13
RPS18
RPS23
RPS10
Module7
PLVAP
SPARCL1
A2M
INS
RAMP2
GNG11
CD52
COL15A1
GIMAP4
RAMP3
Module8
MYL6
MDK
BST2
ARPC2
MARCKS
PFN1
ST14
PYCARD
H1F0
RARRES3
Module9
CEACAM6
TFF1
LGALS4
FXYD3
TFF3
CYP3A5
LSR
LYZ
S100P
CLDN18
Module10
TSPAN8
GPX2
SPINK1
ANXA4
TESC
ARL6IP1
ELF3
OLFM4
ERBB3
HSD17B2
kME
C
Module5
Module1
Module4
Module6
Module7
Module9
Module10
Module8
Module2
Module3
B
KEGG pathways (by Count) in Module1
Cellular senescence
Apoptosis
Cytoskeleton in muscle cells
Lysosome biogenesis
Nucleotide excision repair
Ubiquitin mediated proteolysis
Ribosome
Protein processing in endoplasmic reticulum
Diabetic cardiomyopathy
Salmonella infection
Shigellosis
Parkinson disease
Glycosaminoglycan biosynthesis-hondroitin sulfate/dermatan sulfate
Nucleotide metabolism
p.adjust
Gene Count
KEGG pathways (by Count) in Module8
Cytoskeleton in muscle cellsShigellosis
Regulation of actin cytoskeleton
Focal adhesion
Pertussis
Yersinia infection
Proteoglycans in cancer
Shigellosis
Fc gamma R-mediated phagosome formation
Virion - Hepatitis viruses
Integrin signaling
Gene Count
D
Correlation Distribution of THBS2 (myCAF) with Various Gene Sets
Based on Spearman Correlation Coefficients Between Samples
Kruskal-Wallis p = 3.15e-11
Spearman Correlation
E
THBS2 (myCAF) correlation with hallmark genes (all)
Kruskal-Wallis p = 0.0205
Spearman Correlation
F
Odds Ratio by Module (EMT set: Nichenet)
Fisher's exact test, FDR-adjusted p-value
Odds Ratio by Module (EMT set: pEMT)
Fisher's exact test, FDR-adjusted p-value
Odds Ratio
G
THBS2 (myCAF) correlation with Nichenet genes (all)
Kruskal-Wallis p = 0.0562
THBS2 (myCAF) correlation with Nichenet genes (positive only)
Kruskal-Wallis p = 0.162
THBS2 (myCAF) correlation with pEMT genes (all)
Kruskal-Wallis p = 0.151
THBS2 (myCAF) correlation with pEMT genes (positive only)
Kruskal-Wallis p = 0.161
Spearman Correlation
H
Tumor -vs- TAC in Jiang et al.'s data
logFC:1 ; AdjP:0.05 ; Up:709 ; Down:408
Tumor -vs- TAC in Cao et al.'s Protein data
logFC:1 ; AdjP:0.05 ; Up:127 ; Down:201
Tumor -vs- TAC in Cao et al.'s mRNA data
logFC:1 ; AdjP:0.05 ; Up:1805 ; Down:1584
-log10(adjusted P value)
log2FoldChange
group
up
down
not sig
up_Cao_mRNA
up_Jiang_Protein
up_Cao_Protein
Core genes (23):
S100A11, AHNAK2,COL3A1,COL1A2,
KRT7,KRT19, ITGA2,MX1,FHL2,
P4HA1,COL1A1,SULF1,OAS2,VCAN,
S100A6,SERPINH1,COL12A1,PDLIM7,
S100A14,FN1,POSTN,THBS2,LGALS3BP
I
FN1_ITGA3(minprop=0.10)
Overall P < 0.001
FN1_ITGB1(minprop=0.30)
Overall P = 0.017
FN1_SDC1(minprop=0.30)
Overall P = 0.007
FN1_SDC4 (minprop=0.30)
Overall P = 0.034
High/High
High/Low
Low/High
Low/Low
Survival probability
Months after diagnosis
Number at risk
Strata

Supplementary Figure S6. hdWGCNA identifies THBS2-associated gene modules linked to EMT. (A) Module connectivity within each identified module from hdWGCNA analysis. (B) KEGG pathway enrichment plots for Module 1 and Module 8 (One-tailed hypergeometric test with Benjamini–Hochberg FDR correction). (C) Heatmap of correlation analysis among different gene modules. (D) Spearman's correlation coefficient between *THBS2* expression and overall gene modules. (E) Distribution of Spearman's correlation coefficients between *THBS2* expression and module genes within the Hallmark-EMT gene set. (F) Bar plot showing enrichment levels of the NicheNet-pEMT gene set and pEMT gene set in different gene modules (Fisher's exact test with Benjamini–Hochberg FDR correction). (G) Boxplot showing the distribution of Spearman's correlation coefficients between *THBS2* expression and module genes within the NicheNet-pEMT gene set and pEMT gene set. (H) Volcano plots showing differential expression analysis of genes and proteins in the Cao's and Jiang's datasets; Venn diagram showing the overlap of commonly upregulated differential molecules. (I) Kaplan–Meier survival curves (Cao's cohort protein dataset) showing prognostic differences in patients with high versus low combined expression of FN1-ITGA3, FN1-ITGB1, FN1-SDC1, and FN1-SDC4 (log-rank test). Statistical information: Panel C: two-sided Pearson's correlation coefficient test; Panel D, E and G: two-sided Kruskal-Wallis test. $^{*}p < 0.05$, $^{**}p < 0.01$, $^{***}p < 0.001$; $p \geqslant 0.05$ are left unmarked.

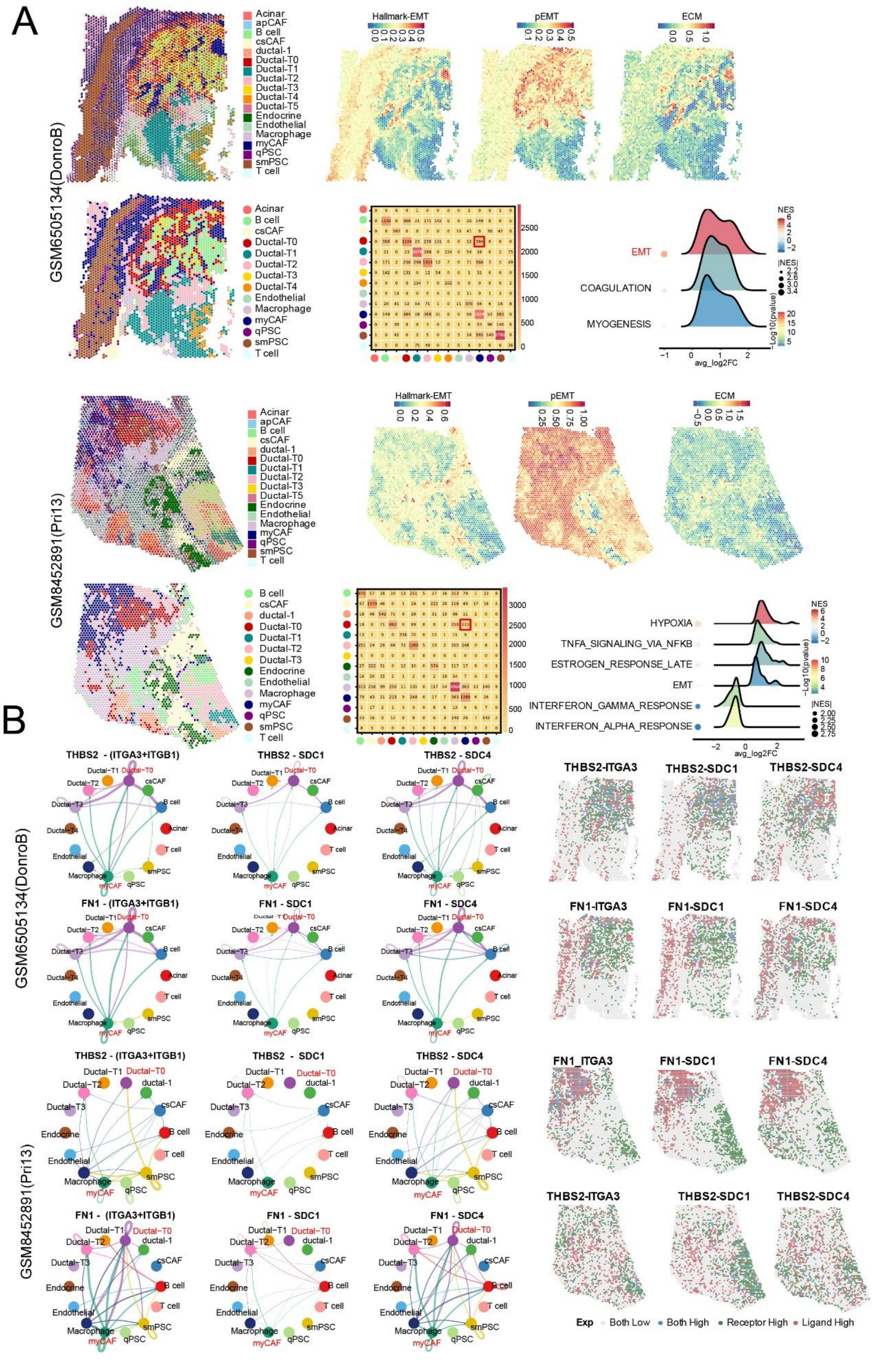
A
GSM6505134(DonroB)
Acinar
apCAF
B cell
csCAF
ductal-1
Ductal-T0
Ductal-T1
Ductal-T2
Ductal-T3
Ductal-T4
Ductal-T5
Endocrine
Endothelial
Macrophage
myCAF
qPSC
smPSC
T cell
Hallmark-EMT
pEMT
ECM
NES
|NES|
-Log10(pvalue)
EMT
COAGULATION
MYOGENESIS
avg_log2FC
GSM8452891(Pri13)
HYPOXIA
TNFA_SIGNALING_VIA_NFKB
ESTROGEN_RESPONSE_LATE
INTERFERON_GAMMA_RESPONSE
INTERFERON_ALPHA_RESPONSE
B
THBS2 - (ITGA3+ITGB1)
THBS2 - SDC1
THBS2 - SDC4
FN1 - (ITGA3+ITGB1)
FN1 - SDC1
FN1 - SDC4
THBS2-ITGA3
THBS2-SDC1
THBS2-SDC4
FN1-ITGA3
FN1-SDC1
FN1-SDC4
FN1_ITGA3
Exp
Both Low
Both High
Receptor High
Ligand High

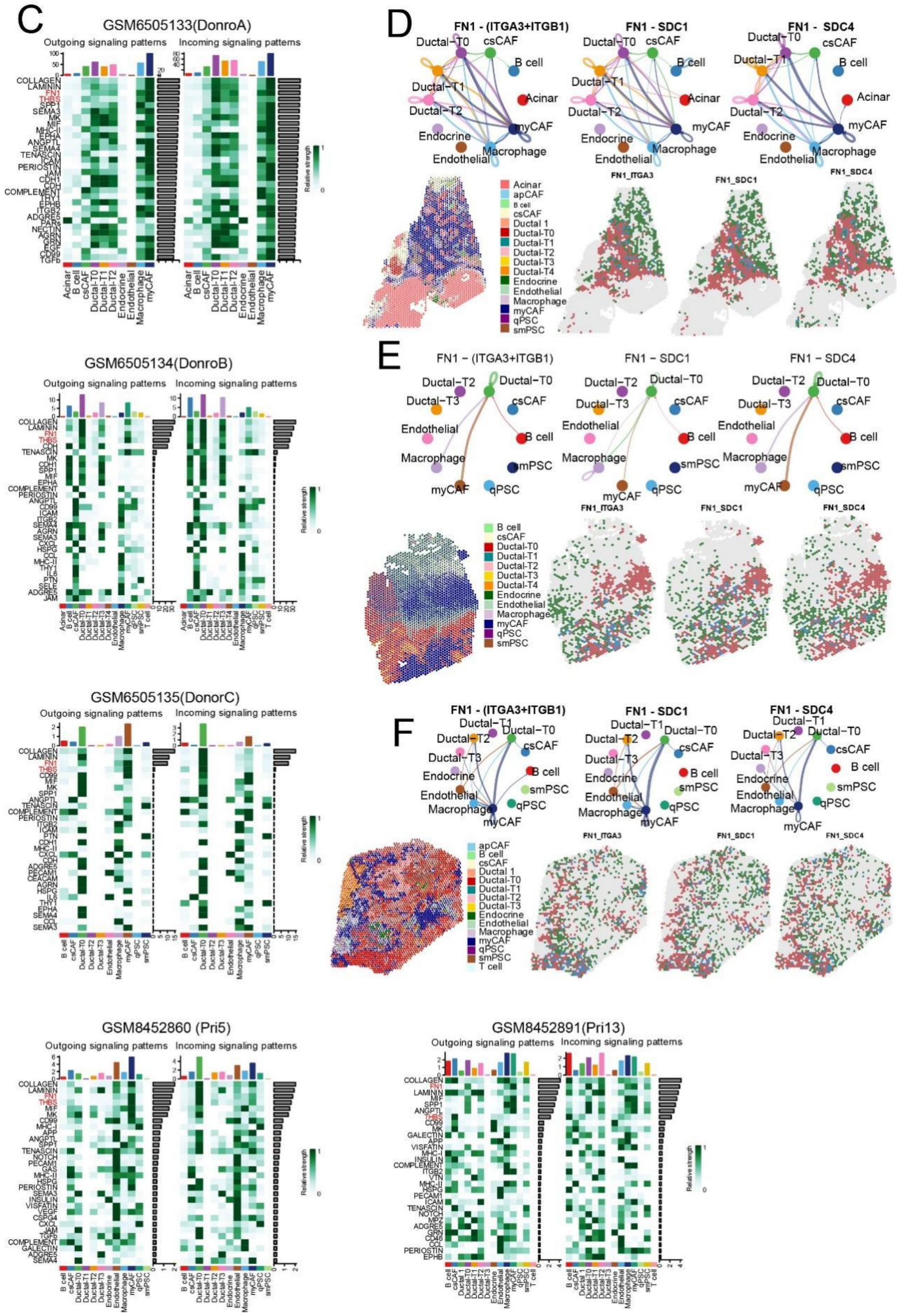
C
GSM6505133(DonroA)
Outgoing signaling patterns
Incoming signaling patterns
COLLAGEN
LAMININ
FN1
THBS
SPP1
SEMA3
MK
MIF
MHC-II
EPHA
ANGPTL
SEMA4
TENASCIN
ICAM
PERIOSTIN
JAM
CDH1
CDH
COMPLEMENT
THY1
EPHB
ITGB2
ADGRE5
NECTIN
AGRN
GRN
EGF
CD99
TGFb
Relative strength
Acinar
B cell
csCAF
Ductal-T0
Ductal-T1
Ductal-T2
Endocrine
Endothelial
Macrophage
myCAF
D
FN1 - (ITGA3+ITGB1)
FN1 - SDC1
FN1 - SDC4
Ductal-T0
csCAF
B cell
Ductal-T1
Acinar
Ductal-T2
myCAF
Endocrine
Macrophage
Endothelial
Acinar
apCAF
B cell
csCAF
Ductal 1
Ductal-T0
Ductal-T1
Ductal-T2
Ductal-T3
Ductal-T4
Endocrine
Endothelial
Macrophage
myCAF
qPSC
smPSC
FN1_ITGA3
FN1_SDC1
FN1_SDC4
GSM6505134(DonroB)
Outgoing signaling patterns
Incoming signaling patterns
E
FN1 - (ITGA3+ITGB1)
FN1 - SDC1
FN1 - SDC4
Ductal-T2
Ductal-T0
Ductal-T3
csCAF
Endothelial
B cell
Macrophage
smPSC
myCAF
qPSC
B cell
csCAF
Ductal-T0
Ductal-T1
Ductal-T2
Ductal-T3
Ductal-T4
Endocrine
Endothelial
Macrophage
myCAF
qPSC
smPSC
FN1_ITGA3
FN1_SDC1
FN1_SDC4
GSM6505135(DonorC)
Outgoing signaling patterns
Incoming signaling patterns
F
FN1 - (ITGA3+ITGB1)
FN1 - SDC1
FN1 - SDC4
Ductal-T1
Ductal-T0
Ductal-T2
Ductal-T3
csCAF
B cell
Endocrine
smPSC
Endothelial
qPSC
Macrophage
myCAF
apCAF
B cell
csCAF
Ductal 1
Ductal-T0
Ductal-T1
Ductal-T2
Ductal-T3
Endocrine
Endothelial
Macrophage
myCAF
qPSC
smPSC
T cell
FN1_ITGA3
FN1_SDC1
FN1_SDC4
GSM8452860 (Pri5)
Outgoing signaling patterns
Incoming signaling patterns
GSM8452891(Pri13)
Outgoing signaling patterns
Incoming signaling patterns

Supplementary Figure S7. Spatial colocalization of myCAFs and Ductal-T0 cells in an EMT- active niche. (A) Spatial maps of tissue sections showing the spatial distribution of major cell type proportions, coloring based on the cell type with the maximum proportion, and the spatial distribution of EMT-related scores; hexagonal neighborhood interaction heatmap; mountain plot of GSEA for differentially expressed genes in myCAFs (Permutation-based significance with Benjamini–Hochberg FDR correction). (B) Cell-cell interaction network of *FN1* and its receptors in the spatial transcriptome inferred by CellChat, with colocalization patterns shown in spatial tissue maps. (C) Heatmap of intercellular communication inferred from spatial transcriptome data, showing signal outgoing (left) and incoming (right) patterns. (D-F) Cell-cell interaction network of *FN1* and its receptors in the spatial transcriptome inferred; spatial tissue maps showing major cell type proportions and ligand-receptor colocalization patterns.

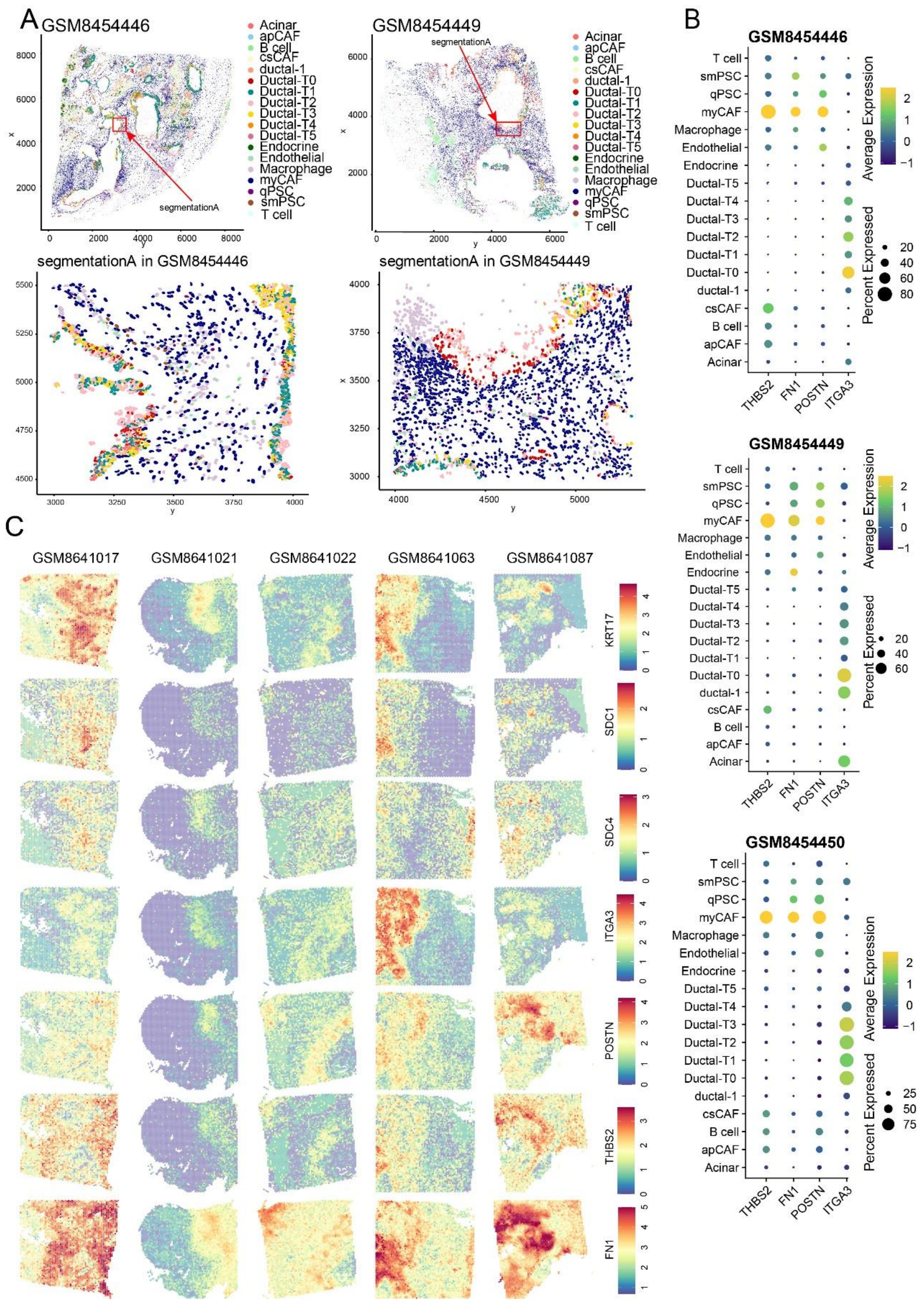
A
GSM8454446
GSM8454449
segmentationA
Acinar
apCAF
B cell
csCAF
ductal-1
Ductal-T0
Ductal-T1
Ductal-T2
Ductal-T3
Ductal-T4
Ductal-T5
Endocrine
Endothelial
Macrophage
myCAF
qPSC
smPSC
T cell
segmentationA in GSM8454446
segmentationA in GSM8454449
B
GSM8454446
GSM8454449
GSM8454450
Average Expression
Percent Expressed
THBS2
FN1
POSTN
ITGA3
C
GSM8641017
GSM8641021
GSM8641022
GSM8641063
GSM8641087
KRT17
SDC1
SDC4
ITGA3
POSTN
THBS2
FN1

Supplementary Figure S8. Colocalization of malignant ductal cells, myCAFs, and THBS2-receptor axes. (A) Overview of two Xenium tissue sections showing major cell types, with magnified insets displaying the spatial arrangement and colocalization of local cell types. (B) Bubble plot showing the expression of *FN1*, *THBS2*, and *ITGA3*. (C) Spatial colocalization of THBS2-SDC1, THBS2-SDC4, and THBS2-ITGA3 using *KRT7* (malignant ductal cells) and *FN1* (myCAFs) as markers in the BMS Spatial Portal for Treatment Naïve PDAC database (https://periscopeapps.org/pdac_spatial_portal/).
